# Single‑cell RNA sequencing analysis of human embryos from the late Carnegie to fetal development

**DOI:** 10.1186/s13578-024-01302-9

**Published:** 2024-09-12

**Authors:** Chengniu Wang, Xiaorong Wang, Wenran Wang, Yufei Chen, Hanqing Chen, Weizhen Wang, Taowen Ye, Jin Dong, Chenliang Sun, Xiaoran Li, Chunhong Li, Jiaying Li, Yong Wang, Xingmei Feng, Hongping Ding, Dawei Xu, Jianwu Shi

**Affiliations:** 1 Institute of Reproductive Medicine, Medical School, Nantong University, Nantong, 226001 Jiangsu China; 2 Basic Medical Research Centre, Medical School, Nantong University, Nantong, 226001 Jiangsu China; 3 Center for Reproductive Medicine, Affiliated Maternity and Child Health Care Hospital of Nantong University, Nantong, 226018 Jiangsu China; 4 Blood Purification Centre, Third People’s Hospital of Rugao, Nantong, 226531 Jiangsu China; 5 Department of Orthopedics, Affiliated Hospital 2 of Nantong University, Nantong, 226000 Jiangsu China; 6 Department of Stomatology, Affiliated Hospital of Nantong University, Medical School of Nantong University, Nantong, 226001 Jiangsu China; 7 Nantong Institute of Genetics and Reproductive Medicine, Affiliated Maternity and Child Healthcare Hospital of Nantong University, Nantong, 226018 Jiangsu China; 8 Nantong Key Laboratory of Genetics and Reproductive Medicine, Nantong, 226018 Jiangsu China; 9 Department of Critical Care Medicine, Affiliated Hospital of Nantong University, Medical School of Nantong University, Nantong, 226001 Jiangsu China; 10 Department of Neurosurgery, Affiliated Hospital 2 of Nantong University, Nantong, 226006 Jiangsu China

**Keywords:** Single-cell RNA sequencing, Human embryos, Cell development, Cell differentiation

## Abstract

**Background:**

The cell development atlas of transition stage from late Carnegie to fetal development (7–9 weeks) remain unclear. It can be seen that the early period of human embryos (7–9 weeks) is a critical research gap. Therefore, we employed single‑cell RNA sequencing to identify cell types and elucidate differentiation relationships.

**Results:**

The single‑cell RNA sequencing analysis determines eighteen cell clusters in human embryos during the 7–9 weeks period. We uncover two distinct pathways of cellular development and differentiation. Initially, mesenchymal progenitor cells differentiated into osteoblast progenitor cells and neural stem cells, respectively. Neural stem cells further differentiated into neurons. Alternatively, multipotential stem cells differentiated into adipocyte, hematopoietic stem cells and neutrophil, respectively. Additionally, COL1A2-(ITGA1 + ITGB1) mediated the cell communication between mesenchymal progenitor cells and osteoblast progenitor cells. NCAM1-FGFR1 facilitated the cell communication between mesenchymal progenitor cells and neural stem cells. Notably, NCAM1-NCAM1 as a major contributor mediated the cell communication between neural stem cells and neurons. Moreover, CGA-FSHR simultaneously mediated the communication between multipotential stem cells, adipocyte, hematopoietic stem cells and neutrophil. Distinct cell clusters activated specific transcription factors such as HIC1, LMX1B, TWIST1, and et al., which were responsible for their specific functions. These coregulators, such as HOXB13, VSX2, PAX5, and et al., may mediate cell development and differentiation in human embryos.

**Conclusions:**

We provide the cell development atlas for human embryos (7–9 weeks). Two distinct cell development and differentiation pathways are revealed.

**Supplementary Information:**

The online version contains supplementary material available at 10.1186/s13578-024-01302-9.

## Background

Researches on embryonic development have predominantly focused model organisms such as mice and zebrafish [1–4]. Single-cell sequencing has established the cell atlas of embryonic development for zebrafish and frog [2, 4, 5]. In mouse embryos, the genomic expression activity of 116,312 single cells (from 6.5 to 8.5 days) is examined. This analysis has provided the gene activation information at various time points within the 48 h of gastrulation formation [3]. Furthermore, a comprehensive atlas of 2 million cells from 61 mouse embryos (from 9.5 to 13.5 days) has been constructed [1]. In Macaca fascicularis, six Carnegie stage embryos (CS8 to CS11) are collected and 56,636 single cells are analyzed. The primary cell types of gastral motor stages including protonogenesis, somatic cell development, intestinal duct formation, neural tube formation and neural crest differentiation are identified [6].

The initial 60 days (8 weeks) of human embryonic development can be categorized into 23 Carnegie stages [7, 8]. The cell differentiation and morphogenesis at various developmental stages can be characterized by the formation of zygote, pronuclei, morula, blastocyst, free blastocyst and the attaching blastocyst (CS1-CS4, Week 1), the implantation and formation of extraembryonic mesoderm, primitive streak, gastrulation (CS5-CS6, Week 2), the development and formation of gastrulation, notochordal process, primitive pit, notochordal canal, somite number 1–3 neural folds, cardiac primordium, head fold (CS7-CS9, Week 3), the somitogenesis of somite number 4–12 neural fold fuses, somite number 13–20 rostral neuropore closes, somite number 21–29 caudal neuropore closes, somite number 30 leg buds, lens placode, pharyngeal arches (CS10-CS13, Week 4), the development and formation of lens pit, optic cup, lens vesicle, nasal pit, hand plate (CS14-CS15, Week 5), the development and formation of nasal pits moved ventrally, auricular hillocks, foot plate, finger rays (CS16-CS17, Week 6), the ossification commences, straightening of trunk (CS18-CS19, Week 7), the upper limbs longer and bent at elbow, hands and feet turned inward, eyelids, external ears, rounded head, body and limbs (CS20-CS23, Week 8) [9]. Stage 23 is the final stage of the embryo, and subsequent development is called fetal development.

At present, the development mechanisms of human embryos from stage CS1 to CS7 (1 day–2.5 weeks) have been analyzed [10–13]. Furthermore, human embryos aged 16 to 19 days have demonstrated differentiation into eleven distinct cell types including progenitor cells of the blood system and primordial germ cell [14]. In some studies, the scRNA-seq combined spatial transcriptome technology is employed to construct the cell atlas of early human embryos from stage CS12 to CS16 (4–6 weeks). Meanwhile, the single cell sequencing data of CS12–CS16 (4–6 weeks) is integrated with the fetal organ data aged 10 to 26 weeks. This analysis has characterized the ancestral state of each fetal organ and facilitated the discovery of previously unknown cell types [15]. Moreover, the gene expression atlas of four million single cells from 121 human fetal samples (72 to 129 days) are constructed using sci-RNA-seq3 [16]. The chromatin accessibility of more than 800,000 fetal single cells is detected using sci-ATAC-seq3. More than one million sites of chromatin accessibility have been found and many of which are only present in certain cells [17].

However, the cell development atlas and differentiation of transition stage from late Carnegie to fetal development (7–9 weeks) have not been reported. It can be seen that the early period of human embryos (7–9 weeks) is a critical research gap that is largely unknown. In this study, we accomplished single-cell sequencing and identified the distinct cell types and their differentiation relationships of human embryos (7–9 weeks). These findings have significant implications not only for the investigation of embryonic cell function and differentiation, but the reconstruction of embryonic cell differentiation trajectory in human being.

## Methods

### Collection of human embryos and preparation of single‑cell suspensions

This study was reviewed and approved by the Institutional Review Boards of the Rugao Third People’s Hospital and Nantong University Hospital (2020-K013). For scRNA-seq, the human embryos (one 7-week-old human embryo, one 8-week-old human embryo and one 9-week-old human embryo) were segmented into pieces (2 × 5 × 5 = 50 mm^3^), respectively. The dissociation of human embryonic cells was performed according to the previous study [18]. The tissue pieces were digested with 2 mL tissue digestible solution at 37 °C for 15 min. The cell debris and other impurities were removed using a 40-μm sterile cell filter. Red blood cell lysate of 2 mL was used to lyse red blood cells at 25 °C for 10 min. The cells were counted using the TC_20_ Automatic Cell Counter (Bio-Rad).

### Library preparation and data processing

The single-cell suspension density was adjusted to 1 × 10^5^ cells/mL. The single-cell suspension was loaded into a microfluidic plate and segmented into a single pore chip. Next, cell barcode beads were introduced into the microchip and cleaned. A single-cell lysis buffer (100 μL) was added to the chip, leading to cell lysis and mRNA capture. The cell barcode beads, along with the captured mRNAs, were ejected from the chip for subsequent reverse transcription, cDNA amplification and library construction. After fragment size selection and purification, 150-bp paired-ended sequencing of the single-cell library was performed by Illumina HiSeq × 10 machine. The data of single cell sequencing was in FASTQ format. CeleScope software package was used for comparison and quantification and gene expression matrix was generated.

### Dimension‑reduction and clustering analysis

The dimension reduction and clustering analysis of the single-cell RNA expression matrix was performed using Seurat package. Each matrix was loaded to create a Seurat object. Low quality cells were filtered using the same criteria for each sample (the number of distinct RNA molecules is between 200 and 6000, the total RNA count is no more than 30,000, and the percentage of mitochondrial should be less than 30%). After quality control, all Seurat objects were merged into a single object. Subsequently, the matrix was normalized using SCTransform algorithm, and 3000 highly variable genes were obtained using FindVariableFeatures function. In addition, the RunPCA function was used for principal component analysis and 50 PCs were retained. The FindClusters and FindNeighbors functions were used for cluster analysis, and the dimension‑reduction of cell clusters was visualized by UMAP method.

### Cell type annotation and enrichment analyses

By referring to the curated marker lists on the CellMarker and PanglaoDB database, the cell clusters were annotated. In addition, the marker genes which were reported in published literatures were also used in the annotation of cell types (Supplemental Material Table 1). The FindAllMarkers function (min.pct = 0.25, logfc.threshold = 0.25) was used to screen differentially expressed genes. Subsequently, the cell type-specific marker genes were illustrated. The proportion of cell clusters was also counted. About the differentially expressed genes, the samples were further divided into three groups (Group 1: 8-week-old vs 7-week-old human embryo, Group 2: 9-week-old vs 8-week-old human embryo, Group3: 9-week-old vs 7-week-old human embryo). The differentially expressed genes of cell clusters in different groups were screened. The cell cluster-specific genes (up-regulated or down-regulated in multiple groups or only in a group) were further screened. In regard to investigate the differentially expressed genes during development, we firstly screened the high expression genes of cell clusters in Group 1, Group 2 and Group 3, respectively. We further intersect the genes that are highly expressed in these three groups. The intersect genes are the high expression genes during development. Finally, clusterProfiler v3.6.1 package was used for the enrichment analysis. Biological process (BP) with p_adj value < 0.05 were defined as significantly enriched.

### Trajectory analysis and RNA velocity

Monocle3 was used to construct the differentiation trajectory of the cell clusters. Genes which were expressed in at least 1% of cells and whose empirical discrete value was greater than the fitting discrete value were selected for pseudotime analysis. DDRTree method was used for dimension‑reduction and the reconstruction of cell differentiation trajectory. For RNA velocity, BAM files containing individual cell populations were converted to LOOM files. LOOM files were then converted into clipped and unclipped matrix files. The results were visualized by UMAP algorithm.

### Cell–cell communication analysis

Cell–cell communication was performed using the CellChat package. The Seurat object was used to create a new CellChat object. The human ligand-receptor database (CellChatDB) provided by CellChat was imported. The over-expressed genes (ligand-receptors) of cell clusters were identified. The over-expressed interactions between over-expressed ligand-receptors were then identified. The probability of intercellular communication was calculated and the communication networks were predicted. The “identifyCommunicationPatterns” function was used to analyze the outgoing communication patterns of secretory cells and the incoming communication patterns of target cells.

### Transcription factor analysis

The transcription factor analysis was conducted using pySCENIC package. Before analysis, the filtered expression matrix (Seurat object) was converted to Loom format using the seurat-disk R package. The subsequent pySCENIC processes consists of four major stages: co-expressed genes were used to infer gene regulatory networks, candidate target genes were filtered according to known transcription factor binding sites, AUC (area under the recovery curve) was calculated according to the enriched gene list, and the AUC matrix was binarized. Module analysis was investigated using the Connection Specificity Index (CSI) parameter which were reported in previous studies [19, 20]. Regulatory networks were analyzed using Cytoscape [21].

### Real-time quantitative PCR assays

Total RNA was extracted from the human embryos (7-week-old, 8-week-old and 9-week-old human embryo, respectively). HiScript II Q RT SuperMix for qPCR (Vazyme Biotech, Nanjing, China) were used to synthesize cDNA. Real-time PCR was performed using AceQ^®^ Universal SYBR^®^ qPCR Master Mix (Vazyme Biotech, Nanjing, China) on the CFX96™ Real-Time PCR System (Bio-Rad, California, America). Primers for the target genes were synthesized by Sangon Biotech (Sangon, Shanghai, China). The sequences of primers are listed in Supplemental Material Table 2. The relative mRNA abundances were calculated and normalized to the mean of β-actin mRNA. 2^−ΔΔCt^ method were used to calculate the fold change of mRNA expression.

## Results

### Clustering analysis of human embryonic cells

**Fig. 1:**
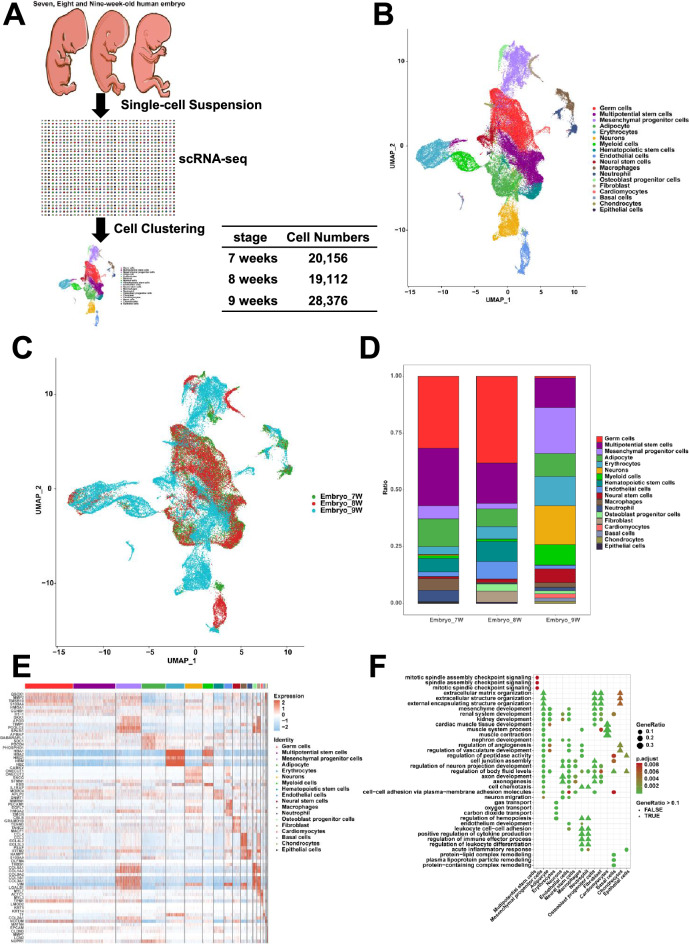
The cell clusters of human embryos (7, 8 and 9 weeks). **A** The flowchart of single‑cell RNA sequencing. **B** The 18 cell clusters in human embryos using UMAP. **C** The 18 cell clusters in 7, 8 and 9-week-old human embryos. **D** The statistics of cell cluster proportions in human embryos (7, 8, 9 weeks). **E** The top five DEGs of cell clusters. **F** GO analysis of cell clusters

The comprehensive cell development atlas of human embryos (20,156 cells from 7-week-old human embryo, 19,112 cells from 8-week-old human embryo and 28,376 cells from 9-week-old human embryo) were constructed (Fig. 1A). After quality control of scRNA-Seq data (Fig. S1A), 67,644 human embryonic cells were clustered into 18 cell clusters. Violin plots showed the expression of MT-ND1, MT-ND2, MT-CO1, MT-CO2, MT-ATP8, MT-ATP6, MT-CO3, MT-ND3, MT-ND4L, MT-ND4, MT-ND5, MT-ND6, MT-CYB in 7-week-old, 8-week-old and 9-week-old human embryo using single cell sequencing data, respectively (Fig. S1B). qPCR confirmed the higher expression of mitochondrial genes in 8-week-old human embryo than that in 7-week-old and 9-week-old human embryo, respectively (Fig. S1C). The 18 cell clusters include germ cells, multipotential stem cells, mesenchymal progenitor cells, adipocyte, erythrocytes, neurons, myeloid cells, hematopoietic stem cells, endothelial cells, neural stem cells, macrophages, neutrophil, osteoblast progenitor cells, cardiomyocytes, fibroblast, basal cells, chondrocytes and epithelial cells (Fig. 1B). The marker genes which were used for annotation were listed (Fig. S2A). The proportions or number of cell clusters were shown (Fig. 1C, D and Fig. S2B, S2C). Violin plots showed the expression of MORC4, APLP2, DNMT1 in hematopoietic stem cells of 7-week-old, 8-week-old and 9-week-old human embryo using single cell sequencing data, respectively (Fig. S2D). qPCR confirmed the lower expression of MORC4, APLP2, DNMT1 in hematopoietic stem cells of 9-week-old human embryo than that in 7-week-old and 8-week-old human embryo, respectively (Fig. S2E). The top 5 DEGs of cell clusters were also listed (Fig. 1E). The GO analysis of cell clusters was presented (Fig. 1F).

### Gene expression changes in human embryo development

**Fig. 2:**
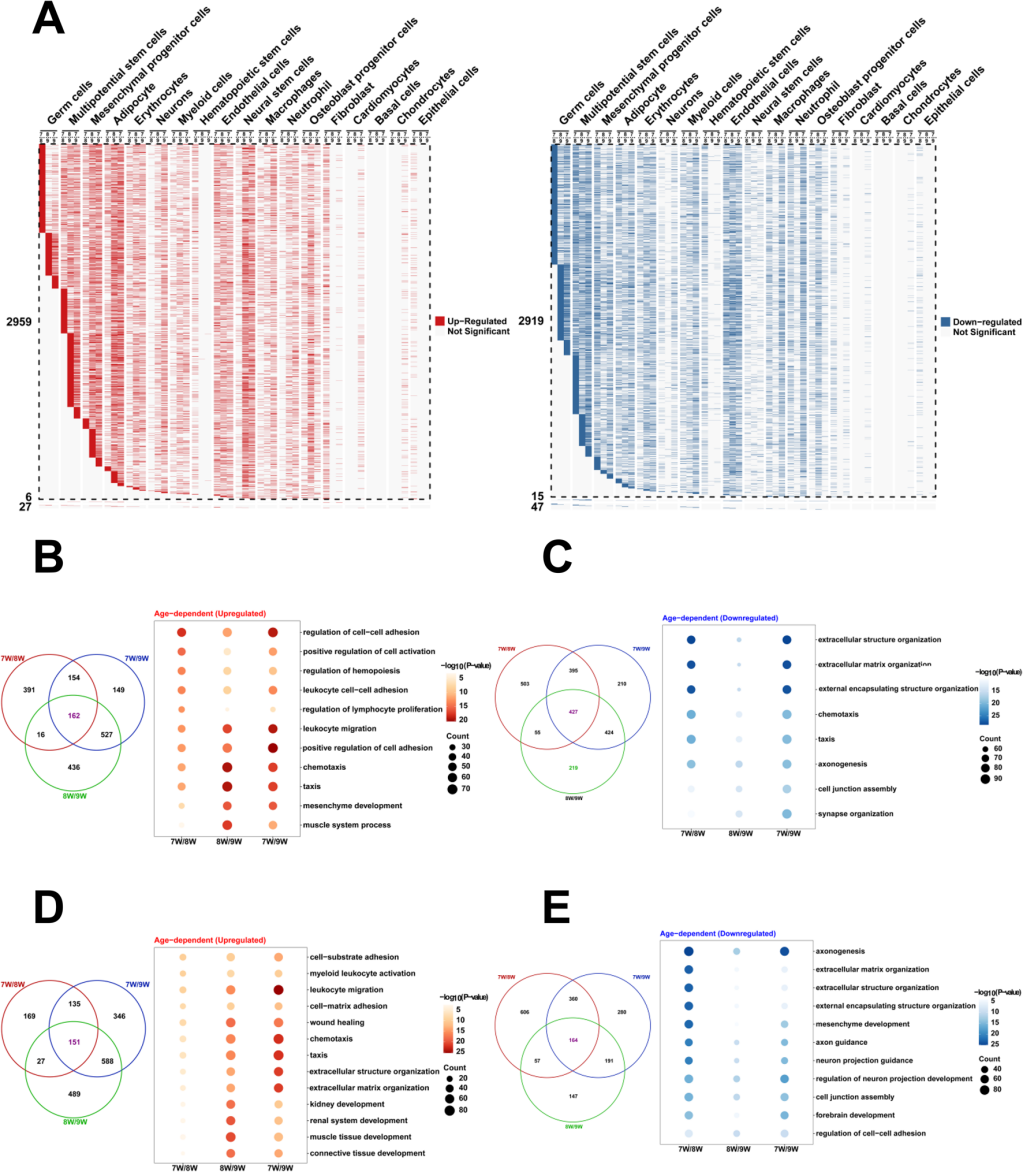
The gene expression of cell clusters during the development of human embryos. **A** The upregulated and downregulated DEGs of cell clusters between different groups (7 and 8 weeks, 8 and 9 weeks, 7 and 9 weeks). **B** Venn showed the number of shared upregulated DEGs of multipotential stem cells and GO enrichment analysis during development. **C** Venn showed the number of shared downregulated DEGs of multipotential stem cells and GO enrichment analysis. **D** Venn showed the number of shared upregulated DEGs of mesenchymal progenitor cells and GO enrichment analysis. **E** Venn showed the number of shared downregulated DEGs of mesenchymal progenitor cells and GO enrichment analysis

On the basis of clustering, we further investigated the gene expression changes of different cell clusters during development. The results showed that the number of upregulated and downregulated genes in germ cells, multipotential stem cells and mesenchymal progenitor cells were dramatic changes during development (Fig. 2A). We further investigated the biological processes of different expression genes (DEGs) of multipotential stem cells and mesenchymal progenitor cells in human embryo development. The results showed that the DEGs of multipotential stem cells participated in the regulation of hemopoiesis, regulation of lymphocyte proliferation, mesenchyme development, leukocyte migration, and et al. (Fig. 2B and C). The results showed that the DEGs of mesenchymal progenitor cells participated in the taxis, kidney development, renal system development, muscle tissue development, and et al. (Fig. 2D and E). These results suggest that these two cell clusters have multifunctional differentiation potentials in human embryo development.

### Cell differentiation relationships analysis using Monocle3 and RNA velocity

**Fig. 3:**
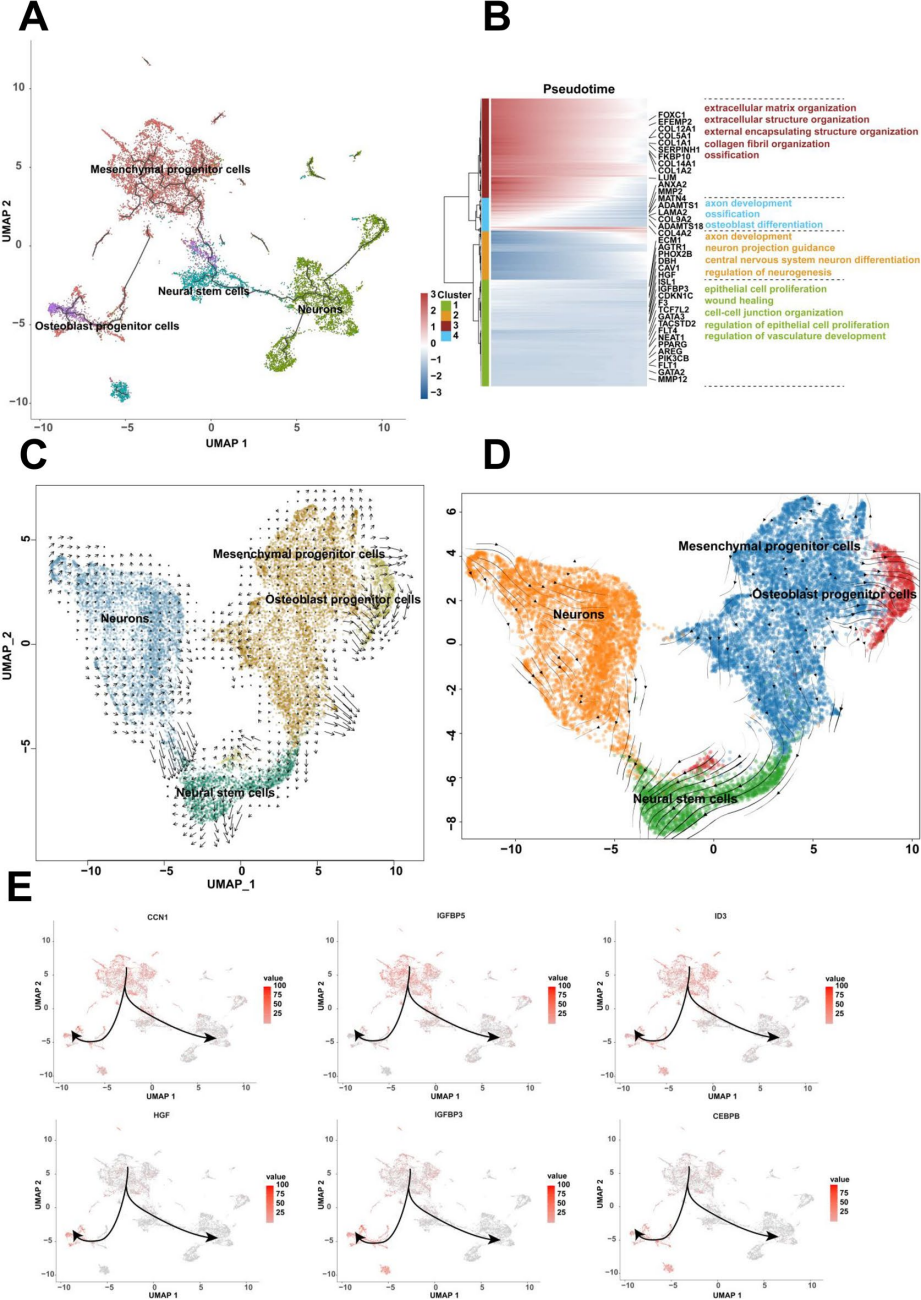
The differentiation relationship of mesenchymal progenitor cells, osteoblast progenitor cells, neural stem cells and neurons. **A** The differentiation relationship among mesenchymal progenitor cells, osteoblast progenitor cells, neural stem cells and neurons using Monocle3. **B** The enrichment analysis of the expression genes along the pseudotime of these four clusters. **C** The differentiation relationship analysis using RNA velocity. **D** The differentiation relationship analysis using scVelo. **E** The expression of CCN1, IGFBP5, ID3, HGF, IGFBP3 and CEBPB among the four clusters

The differentiation relationships among human embryonic cell clusters were calculated by Monocle3. The cell differentiation relationships were found in mesenchymal progenitor cells, osteoblast progenitor cells, neural stem cells and neurons. The mesenchymal progenitor cells were designated as the root in the differentiation pathway. Mesenchymal progenitor cells differentiated into osteoblast progenitor cells and neural stem cells, respectively. Neural stem cells further differentiated into neurons (Fig. 3A). The differentiation extent and orientation of mesenchymal progenitor cells, osteoblast progenitor cells, neural stem cells and neurons were investigated using RNA velocity. The multi-direction arrows showed that mesenchymal progenitor cells and neural stem cells were highly heterogeneous and could differentiate into each direction. The arrow directions of the osteoblast progenitor cells and neurons were consistent, indicating osteoblast progenitor cells and neurons had stable states and differentiated from mesenchymal progenitor cells and neural stem cells, respectively (Fig. 3C and D). The expression genes along the pseudotime of mesenchymal progenitor cells, osteoblast progenitor cells, neural stem cells and neurons could be enriched for the biological process of ossification differentiation, axon development, extracellular matrix organization, and et al. (Fig. 3B). The expression of genes including CCN1, IGFBP5, ID3, HGF, IGFBP3, and CEBPB between mesenchymal progenitor cells and osteoblast progenitor cells were investigated (Fig. 3E). The expression of genes including MAP2, UCHL1, TUBB2B, GAP43, CHL1, and DPYSL2 associated with mesenchymal progenitor cells and neural stem cells were also investigated (Fig. S3A).

**Fig. 4:**
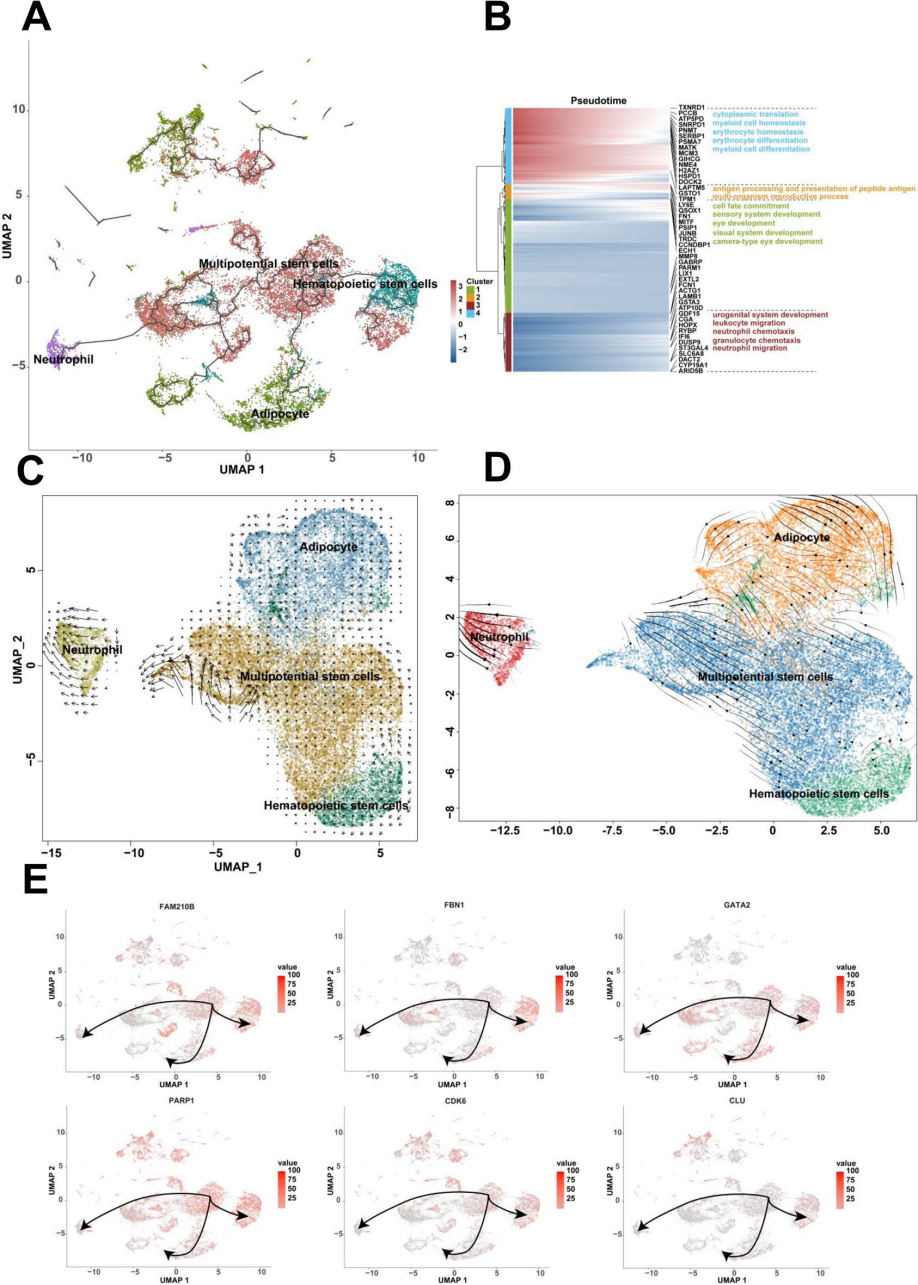
The differentiation relationship of multipotential stem cells, adipocyte, hematopoietic stem cells and neutrophil. **A** The differentiation relationship among multipotential stem cells, adipocyte, hematopoietic stem cells and neutrophil using Monocle3. **B** The enrichment analysis of the expression genes along the pseudotime of these four clusters. **C** The differentiation relationship analysis using RNA velocity. **D** The differentiation relationship analysis using scVelo. **E** The expression of FAM210B, FBN1, GATA2, PARP1, CDK6 and CLU among the four clusters

Another differentiation pathway could be found between multipotential stem cells, adipocyte, hematopoietic stem cells and neutrophil. The multipotential stem cells were designated as the root in this differentiation pathway. Multipotential stem cells differentiated into adipocyte, hematopoietic stem cells and neutrophil, respectively (Fig. 4A). The multi-direction arrows showed that multipotential stem cells were highly heterogeneous and could differentiate into each direction. The arrow directions of the adipocyte, hematopoietic stem cells and neutrophil were consistent, indicating that adipocyte, hematopoietic stem cells and neutrophil had stable states and differentiated from multipotential stem cells (Fig. 4C and D). The expression genes along the pseudotime of multipotential stem cells, adipocyte, hematopoietic stem cells and neutrophil could be enriched for the biological process of myeloid cell homeostasis, erythrocyte differentiation, neutrophil migration, and et al. (Fig. 4B). The expression of genes including FAM210B, FBN1, GATA2, PARP1, CDK6, and CLU associated with multipotential stem cells and hematopoietic stem cells were investigated (Fig. 4E). The expression of genes including PPIB, C1QBP, MDK, LGALS3, LBR and FCER1G associated with multipotential stem cells and neutrophil were also investigated (Fig. S3B).

### Analysis of cell–cell communication using CellChat

**Fig. 5:**
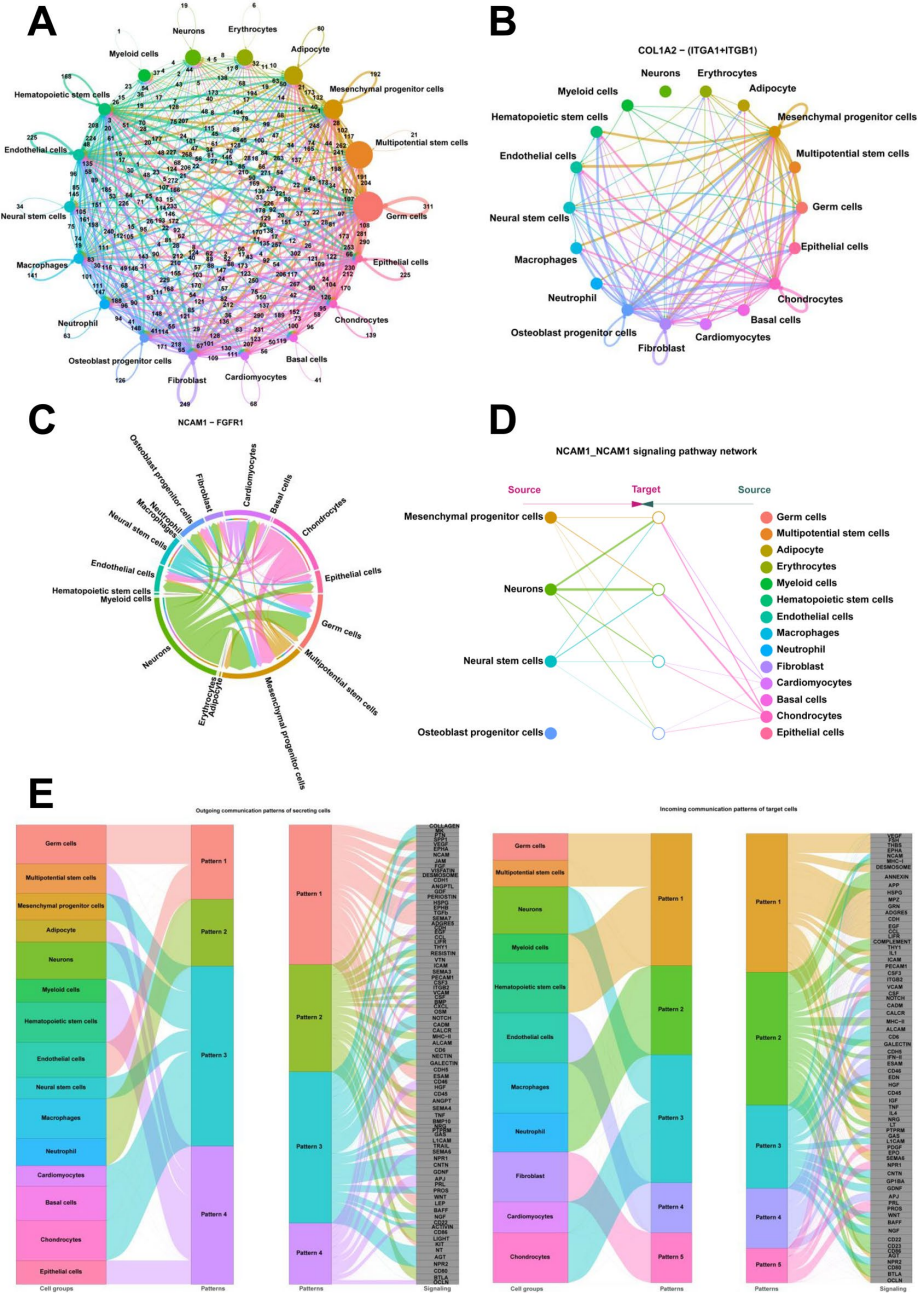
Cell–Cell communication analysis using CellChat. **A** The number of ligand-receptors in each cell cluster. **B** COL1A2-(ITGA1 + ITGB1) signaling pathway in each cell cluster. **C** NCAM1-FGFR1 signaling pathway in each cell cluster. **D** NCAM1-NCAM1 signaling pathway in each cell cluster. **E** The outgoing and incoming communication patterns of each cell cluster

The number and strength of cell–cell interactions across 18 cell clusters was identified using CellChat (Figs. 5A and S4A). The strength of cell–cell interactions across 18 cell clusters in different development stages was also identified (Fig. S5A). The number of ligand-receptors of mesenchymal progenitor cells with other cell clusters were shown in Fig. S4B. Notably, the COL1A2-(ITGA1 + ITGB1) signaling pathway mediated the cell–cell communication between mesenchymal progenitor cells and osteoblast progenitor cells (Figs. 5B and S5B). COL1A2 was found to be highly expressed in mesenchymal progenitor cells and ITGA1 + ITGB1 in osteoblast progenitor cells (Fig. S4C). The number of ligand-receptors of multipotential stem cells with other cell clusters was shown in Fig. S4D. NCAM1-FGFR1 was highly enriched in mesenchymal progenitor cells and neural stem cells (Figs. 5C and S5C). NCAM1-NCAM1 mediated the cell communication between neural stem cells and neurons (Figs. 5D and S5D). The signaling pathways among the patterns of outgoing communication in secretory cells were identified. Mesenchymal progenitor cells, neural stem cells and neurons belong to pattern 3. Pattern 3 contained COLLAGEN, MK, PTN, NCAM, PERIOSTIN, THY1, VTN, SEMA3, VCAM, NOTCH, CADM, ALCAM, CD6, NECTIN, NRG, L1CAM, NPR1, CNTN, GDNF, PROS, NGF, ACTIVIN, NT, AGT and NPR2. Among the patterns of incoming communication of target cells, multipotential stem cells and hematopoietic stem cells belong to pattern 1. Pattern 1 contained VEGF, FSH, THBS, EPHA, DESMOSOME, HSPG, MPZ, GRN, ADGRE5, CDH, EGF, CCL, LIFR, IL1, CSF3, HGF, IGF, IL4, NRG, EPO, SEMA6, AGT and OCLN (Fig. 5E). In this study, CGA-FSHR signaling pathway was found to mediate the cell communication between multipotential stem cells, adipocyte, hematopoietic stem cells and neutrophil (Figs. S4E and S5E). We also found that NAMPT-(ITGA5 + ITGB1) signaling pathway mediated the cell communication between multipotential stem cells and neutrophil (Figs. S4F and S5F).

### Analysis of transcription factors in cell clusters

**Fig. 6:**
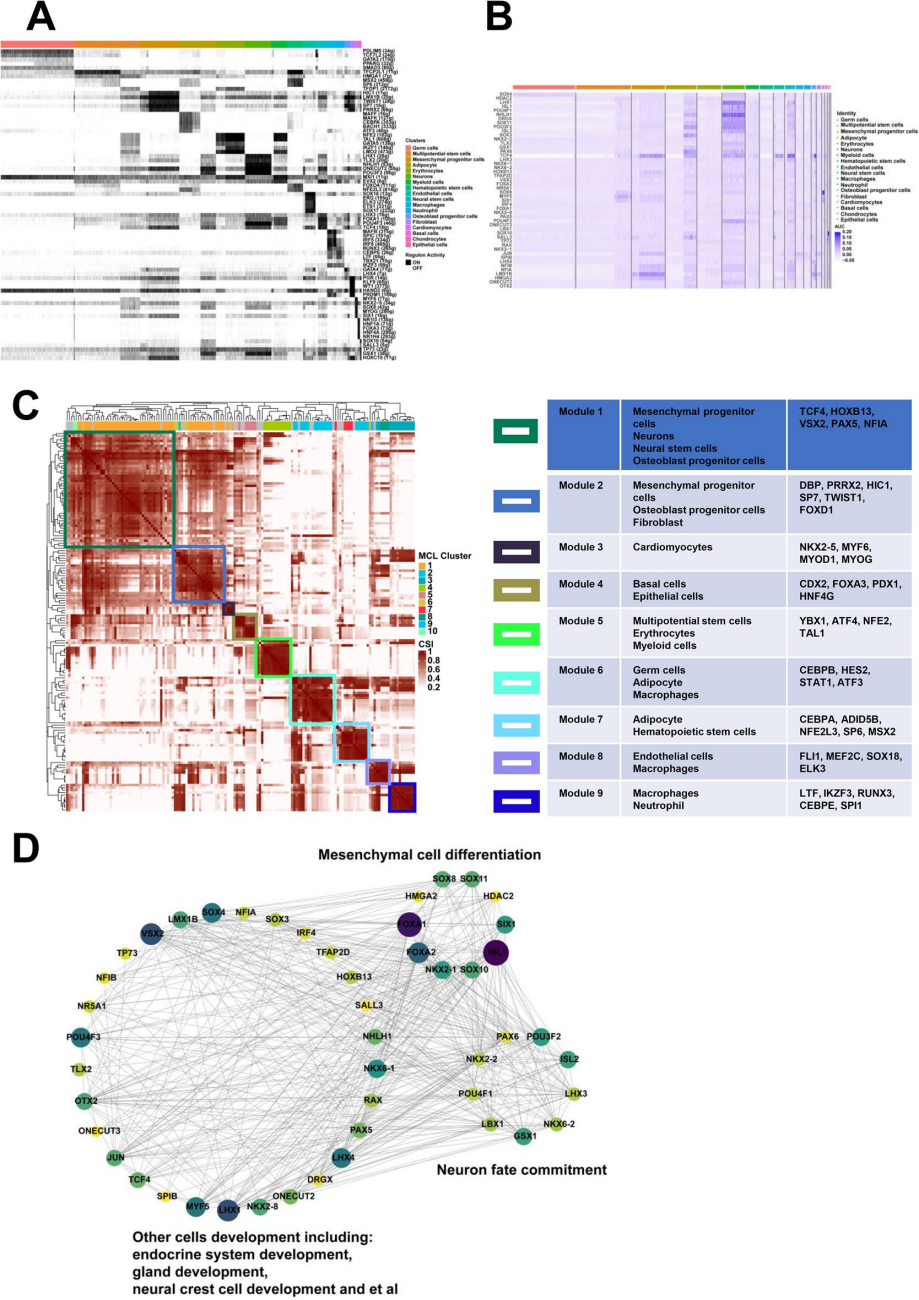
Transcription factor analysis of each cell cluster. **A** The regulons which specifically turned on in cell clusters. **B** Common regulons which turned on between mesenchymal progenitor cells, osteoblast progenitor cells, neural stem cells and neurons. **C** Modules analysis of each cell cluster regulon. **D** Interaction networks showed common regulons between mesenchymal progenitor cells, osteoblast progenitor cells, neural stem cells and neurons

The activity of binary regulators was represented in Fig. 6A. HIC1, LMX1B, TWIST1, SP7 and PRRX2 were turned on in mesenchymal progenitor cells. LHX3, EVX2, FOXA1, POU4F3 and TCF4 were turned on in neural stem cells. LHX1, TLX2, NHLH1, ONECUT2 and POU3F2 were turned on in neurons. GATA4, SP7, LMX1B, TWIST1 and LHX4 were turned on in osteoblast progenitor cells. Furthermore, 152 regulons were clustered into nine modules. The cell clusters and regulons of each module were shown. Module 1 included mesenchymal progenitor cells, neurons, neural stem cells and osteoblast progenitor cells, containing the regulons of TCF4, HOXB13, VSX2, PAX5 and NFIA. Module 2 was organized by mesenchymal progenitor cells, osteoblast progenitor cells and fibroblast, including DBP, PRRX2, HIC1, SP7, TWIST1 and FOXD1. Module 9 consisted of macrophages and neutrophil, containing the regulons of LTF, IKZF3, RUNX3, CEBPE and SPI1 (Fig. 6C). The heatmap showed that common regulators were turned on among mesenchymal progenitor cells, neurons, neural stem cells and osteoblast progenitor cells (Fig. 6B). Interaction mapping of transcription factor networks showed that SOX8, SOX11, HDAC2, SIX1, ISL1, SOX10, NKX2-1, FOXA2, FOXA1 and HMGA2 were important regulators of mesenchymal cell differentiation and were commonly switched on among four cell clusters. In addition, PAX6, POU3F2, ISL2, LHX3, NKX6-2, GSX1, LBX1, POU4F1 and NKX2-2 were associated with neuron fate commitment and also commonly switched on. Finally, other common regulators including SOX4, VSX2, POU4F3, NHLH1, LHX4, TCF4, MYF5, ONECUT2, and et al. were involved in the multi-tissue development including endocrine system development, gland development and neural crest cell development, and et al. (Fig. 6D).

## Discussion

The human embryonic development stage (7–9 weeks) is the important transition stage which is from the late Carnegie to fetal development. However, the cell atlas and differentiation of embryonic development stage (7–9 weeks) have not been reported. In this study, we have constructed the cell development atlas of human embryos (7–9 weeks) using single-cell RNA sequencing. This study fills the research gap from the late Carnegie to fetal development, which has important significance for investigating the development and differentiation relationship of human embryonic cells during the transition stage.

In this work, we identified multipotential stem cells and mesenchymal progenitor cells in human embryos. These two cell clusters possessed multiple differentiation potentials in the development of human embryos. Based on this prediction, the differentiation relationship of multipotential stem cells, mesenchymal progenitor cells and other cell clusters were performed using Monocle 3. The differentiation pathways involved in mesenchymal progenitor cells were identified by trajectory and RNA velocity analysis. Mesenchymal progenitor cells differentiated into osteoblast progenitor cells and neural stem cells, respectively. Neural stem cells could further differentiate into neurons (Fig. 3A). Many investigations show that the osteoblast progenitor cells differentiate from mesenchymal progenitor cells [22–25]. Human primitive mesenchymal stem cells could differentiate into neural stem cells [26]. The trans-differentiation of MSCs into neural lineages could be performed by adding UDP-4 which increases the expression of neural genes and protein [27]. MSCs containing NSC-like cells are reported to differentiate into NSC-like cells [28]. Neural stem cells are responsible for restoring the central nervous system and differentiate into neurons [28]. These results show that the potential differentiation relationships of mesenchymal progenitor cells, neurons, neural stem cells and osteoblast progenitor cells in previous studies. Similar differentiation relationships are also observed in this study. In addition, we also found that the multipotential stem cells could further differentiate into adipocyte, hematopoietic stem cells and neutrophil, respectively (Fig. 4A). In this study, although the differentiation relationships of cell clusters are proposed, the important roles and differentiation of cell clusters need to be further verified. But, on the whole, we have identified high expression genes and differentiation relationships of multipotential stem cells and mesenchymal progenitor cells in human embryos. These results lay a foundation for cell sorting and enrichment of multipotential stem cells and mesenchymal progenitor cells. These results also provide a theoretical basis for in vitro induction of osteoblast progenitor cells, neurons, adipocyte, hematopoietic stem cells and neutrophil. It also provides scientific basis for cell replacement therapy of related cell degenerative diseases.

Cell–cell communication among different cell clusters was performed using CellChat. COL1A2 is the osteoblast differentiation factor and participates in osteoblast maturation [29]. The reduction expression of COL1A2 is found to impact the differentiation of mesenchymal cells into osteoblasts [30]. Integrin α1 (ITGA1) and integrin β1 (ITGB1) play important roles in tumorigenesis, progression and metastasis [31, 32]. In this study, the high expression of COL1A2 in mesenchymal progenitor cells and the high expression of ITGA1 and ITGB1 in osteoblast progenitor cells were observed (Fig. S4C). COL1A2-(ITGA1 + ITGB1) signaling pathway were found to mediate the cell–cell communication between mesenchymal progenitor cells and osteoblast progenitor cells (Fig. 5B). These results show that the important roles of COL1A2-(ITGA1 + ITGB1) signaling pathway in the differentiation process from mesenchymal progenitor cells to osteoblast progenitor cells. NCAM1 (Neural cell adhesion molecule 1) is expressed in neural or mesenchymal stem cells [33, 34]. NCAM1 is reported to play important roles in neuronal migration, axonal branching and synaptogenesis [35]. NCAM1 associates with fibroblast growth factor receptor-1 (FGFR1) [36]. NCAM and FGFR are involved in epithelial-mesenchymal transformation [37]. In this study, NCAM1-FGFR1 signaling pathway was found to mediate the cell–cell communication between mesenchymal progenitor cells and neural stem cells and NCAM1-NCAM1 signaling pathway mediated the cell–cell communication between neural stem cells and neurons (Fig. 5C and D). These results show that the potential important roles of NCAM1 signaling pathway in the differentiation process from mesenchymal progenitor cells to neurons. CGA-FSHR signaling pathway are reported to play important roles in the male reproduction [38], oogenesis [39] and the maintain of secondary sexual characteristics [40]. In this study, CGA-FSHR signaling pathway were involved in the communication between multipotential stem cells, adipocyte, hematopoietic stem cells and neutrophil (Fig. S4E). These results show that the multipotential roles of CGA-FSHR signaling pathway in the differentiation process from multipotential stem cells to other cells. NAMPT signaling pathway is involved in the maturation and survival of neutrophil [41]. In this study, we found that NAMPT-(ITGA5 + ITGB1) signaling pathway mediated the communication between multipotential stem cells and neutrophil (Fig. S4F). These results show that the potential important roles of NAMPT-(ITGA5 + ITGB1) signaling pathway in the differentiation process from multipotential stem cells to neutrophil in human embryo development. We also found other signaling pathways among mesenchymal progenitor cells, neural stem cells and neurons, including COLLAGEN, MK [42], PTN [43], PERIOSTIN [44], THY1, VTN, SEMA3 [45], VCAM, NOTCH [46], CADM, ALCAM, CD6, NECTIN [47], NRG [48], L1CAM [49], NPR1 [50], CNTN, GDNF [51], NGF [52], ACTIVIN [53], NT [54], AGT, NPR2 (Fig. 5E). These reported signaling pathways may be involved in the differentiation of mesenchymal progenitor cells, neural stem cells and neurons in human embryos. The signaling pathways which mediated cell communication in multipotential stem cells and hematopoietic stem cells, including VEGF [55], FSH [56], EPHA [57], DESMOSOME, HSPG, MPZ, GRN, ADGRE5, CDH [58], EGF [59], CCL, LIFR, IL1, CSF3 [60], HGF [61], IGF [62], IL4, NRG, EPO [63], SEMA6, AGT [64] and OCLN (Fig. 5E) were also screened in this study. The roles of these signaling pathways need to be further explored. Therefore, we have identified many known and unknown signaling pathways and molecules that regulate cell differentiation and development in human embryos, which provide targeted pathways or molecules for human embryo cell engineering and in vitro tissue engineering. In subsequent studies, we can achieve cell induction differentiation through the regulation of relevant signaling pathways or molecules.

The specific transcription factors which activated in distinct cell clusters were revealed in this study (Fig. 6A). For example, HIC1, LMX1B, TWIST1, SP7 and PRRX2 were turned on in mesenchymal progenitor cells in this study. HIC1 [65] transforms mesenchymal stem cells (MSCs) into tumor stem cells. LMX1B [66] is involved in the migration and specification of neural crest-derived mesenchymal progenitor cells. TWIST1 [67] and PRRX2 [68] are turned on in mesenchymal progenitor cells. LHX3, EVX2, FOXA1, POU4F3 and TCF4 were turned on in neural stem cells in this study. LHX3 [69] directs the specification of spinal motor neurons (MNs) in embryos. EVX2 [70] specifies the neurotransmitter fates of particular neurons. FOXA1 [71] promotes the differentiation of neural stem-like cells. TCF4 [72] participates in the nervous system development and neural precursor proliferation. LHX1, TLX2, NHLH1, ONECUT2 and POU3F2 were turned on in neurons in this study. LHX1 [73] is required for the maintenance and regeneration of serotonergic neurons in planarians. TLX2 [74] is identified as an important regulator in cortical interneuron development. NHLH1 [75] is involved in neuronal differentiation. ONECUT2 [76] plays an important role in maintaining neuronal characteristics. POU3F2 [77] participates in neuronal differentiation. GATA4, SP7, LMX1B, TWIST1 and LHX4 were turned on in osteoblast progenitor cells in this study. GATA4 [78] regulates osteoblast differentiation and mineralization. SP7 [79] is involved in osteogenic and chondrogenic differentiation from MSCs. TWIST1 [80] is involved in the osteogenic differentiation of MSCs. LHX4 was turned on in osteoblast progenitor cells in this study. These findings suggest that the transcription factors which specifically turned on in distinct cell clusters are responsible for the specific function of each cell cluster. Additionally, the common regulons were turned on in different cell clusters using module analysis (Fig. 6C). For example, module 1 consisted of mesenchymal progenitor cells, neurons, neural stem cells and osteoblast progenitor cells, containing TCF4 [72] which participated in the nervous system development and neural precursor proliferation, HOXB13 [81] which was involved in stem cell differentiation, VSX2 [82] which was a determinant for the fate specification of spinal V2a interneurons, PAX5 and NFIA. Module 2 was organized by mesenchymal progenitor cells, osteoblast progenitor cells and fibroblast, including DBP, PRRX2 [83] which participated in mesenchymal cell differentiation, HIC1 [65] which transformed mesenchymal stem cells into tumor stem cells, SP7 [79] which was involved in osteogenic and chondrogenic differentiation from MSCs, TWIST1 and FOXD1 [84] which was a regulator of osteoblast differentiation. Finally, the interaction networks of co-expression transcription factors showed the complex and robust interaction relationships in mesenchymal progenitor cells, neurons, neural stem cells and osteoblast progenitor cells. The enrichment results of common transcription factors showed that the co-expression transcription factors participated in mesenchymal cell differentiation, neuron fate commitment and multi-tissue development including endocrine system development, gland development and neural crest cell development (Fig. 6D). These co-regulators which turned on between mesenchymal progenitor cells, osteoblast progenitor cells, neural stem cells and neurons may mediate the differentiation of mesenchymal progenitor cells to osteoblast progenitor cells and neural stem cells which can further differentiate into neurons. The samples we collected were in the transitional stage of Carnegie stage to fetal development. Therefore, those transcription factors which were specifically turned on in distinct cell clusters play important roles in the stem maintenance and function of related cell clusters. However, the co-expressed transcription factors are vital for the differentiation among correlated cell clusters.

## Conclusions

**Fig. 7:**
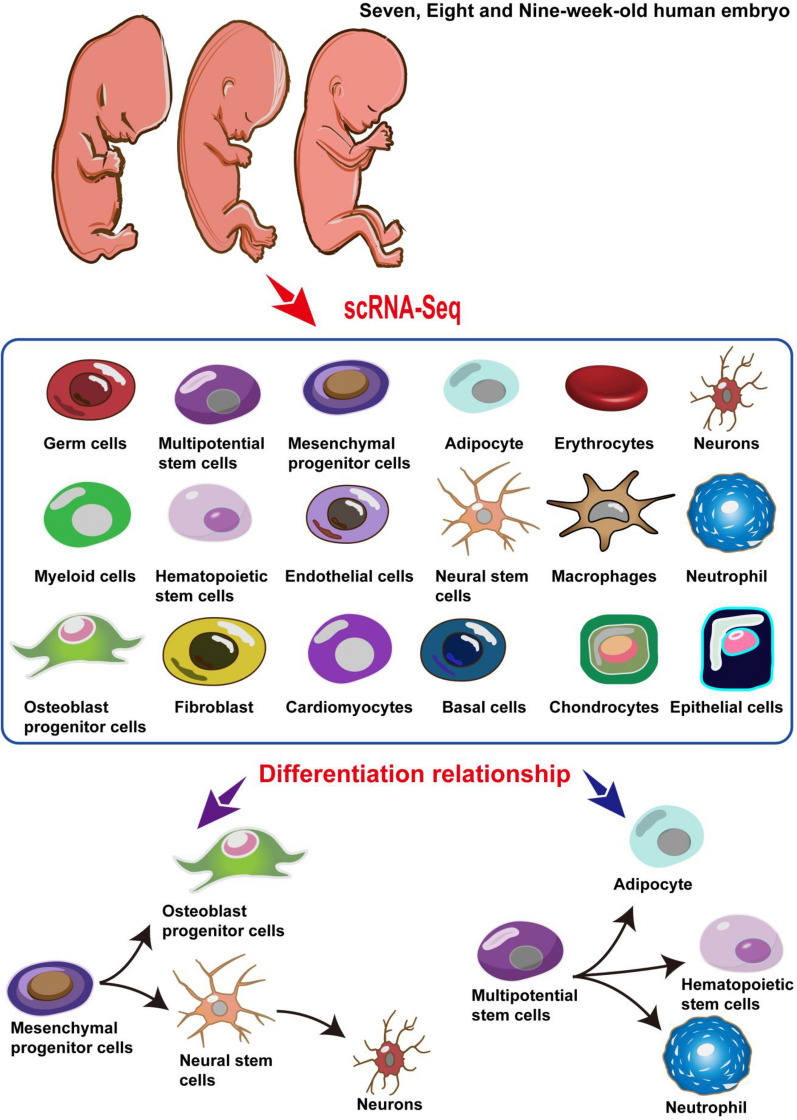
Description of the cell clusters and differentiation relationship of human embryos (7–9 weeks)

In this study, we construct the cell development atlas of human embryos (7–9 weeks). It reveals eighteen distinct cell clusters and two pathways for cell development and differentiation. The potential differentiation relationships and mechanisms between mesenchymal progenitor cells, osteoblast progenitor cells, neural stem cells and neurons are determined. The differentiation relationships and mechanisms of multipotential stem cells, adipocyte, hematopoietic stem cells and neutrophil are also identified (Fig. 7). Although these differentiation relationships and mechanisms need to be further investigating, this study fills the research gap of the cell development atlas and differentiation relationships from the late Carnegie to fetal development. In the future study, the comprehensive cell atlas of the human embryonic cell during embryogenesis could be constructed based on this work and available public data. Our findings provide indispensable resources for investigating the development and differentiation of human embryos. This research also has important significance for the mechanism investigation of aborted embryos, which are caused by abnormal development of embryonic cells.

## Supplementary Information

Additional file 1: **Fig. S1.** Quality control and expression of mitochondrial genes. **A** The number of genes, counts and percentage of mitochondria. **B** Violin plots showing the expression of mitochondrial genes including MT-ND1, MT-ND2, MT-CO1, MT-CO2, MT-ATP8, MT-ATP6, MT-CO3, MT-ND3, MT-ND4L, MT-ND4, MT-ND5, MT-ND6, MT-CYB in the different development stage of human embryos using single cell sequencing data. **C** qPCR validated the expression of mitochondrial genes in different development stages. **Fig. S2.** Marker genes and cell proportion analysis. **A** The marker genes which were screened from the published literatures. **B** The cell proportions of mesenchymal progenitor cells, osteoblast progenitor cells, neural stem cells and neurons. **C** The cell proportions of multipotential stem cells, adipocyte, neutrophil and the number of hematopoietic stem cells.** D** Violin plots showing the expression levels of MORC4, APLP2, DNMT1 in hematopoietic stem cells of different development stage of human embryos using single cell sequencing data. **E** qPCR validated the expression of MORC4, APLP2, DNMT1 in the different development stage of human embryos. **Fig. S3.** The expression of key genes along the pseudotime. **A** The expression of key genes including MAP2, UCHL1, TUBB2B, GAP43, CHL1 and DPYSL2 between mesenchymal progenitor cells and neural stem cells. **B** The expression of PPIB, C1QBP, MDK, LGALS3, LBR and FCER1G between multipotential stem cells and neutrophil. **Fig. S4.** Cell–Cell communication analysis of each cell cluster. **A** The interaction strength of ligand-receptors of each cell cluster. **B** The number of ligand-receptors of mesenchymal progenitor cells with other cell clusters. **C** The expression of COL1A2, ITGA1 and ITGB1 among 18 cell clusters. **D** The number of ligand-receptors of multipotential stem cells with other cell clusters. **E** CGA-FSHR signaling pathway in each cell cluster. **F** NAMPT-signaling pathway in each cell cluster. **Fig. S5.** The strength and signaling pathways of cell clusters in the different development stage of human embryos using single cell sequencing data. **A** The interaction strength of cell clusters in different development stages. **B** COL1A2-signaling pathway of cell clusters in different development stages. **C** NCAM1-FGFR1 signaling pathway of cell clusters in different development stages. **D** NCAM1-NCAM1 signaling pathway of cell clusters in different development stages. **E** CGA-FSHR signaling pathway of cell clusters in different development stages. **F** NAMPT-signaling pathway of cell clusters in different development stages.

Additional file 2: **Table S1.** Marker genes for annotation of human embryo cell clusters.

Additional file 3: **Table S2.** Primers for quantitative RT-PCR.

## Data Availability

The raw data for this study are deposited in the Genome Sequence Archive [85] at the National Genomics Data Center for Biological Information/National Genomics Data Center [86], Institute of Genomic Research, Chinese Academy of Sciences, Beijing, China (GSA-Human: HRA005835).
