# Supplementary figures and images for: Single‑cell RNA sequencing analysis of human embryos from the late Carnegie to fetal development
Source: Cell Biosci. 2024 Sep 12;14:118. doi: 10.1186/s13578-024-01302-9 (PMC11395182; doi:10.1186/s13578-024-01302-9)

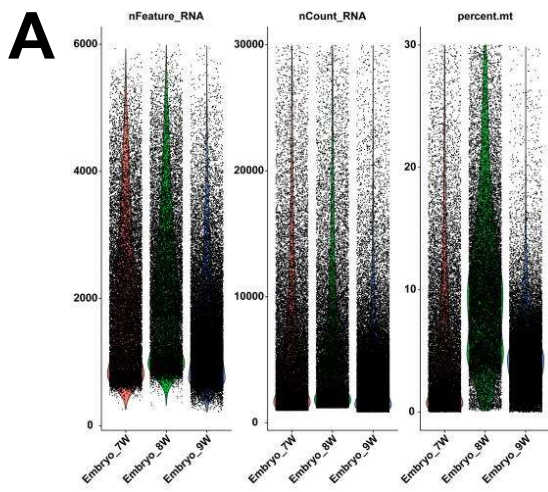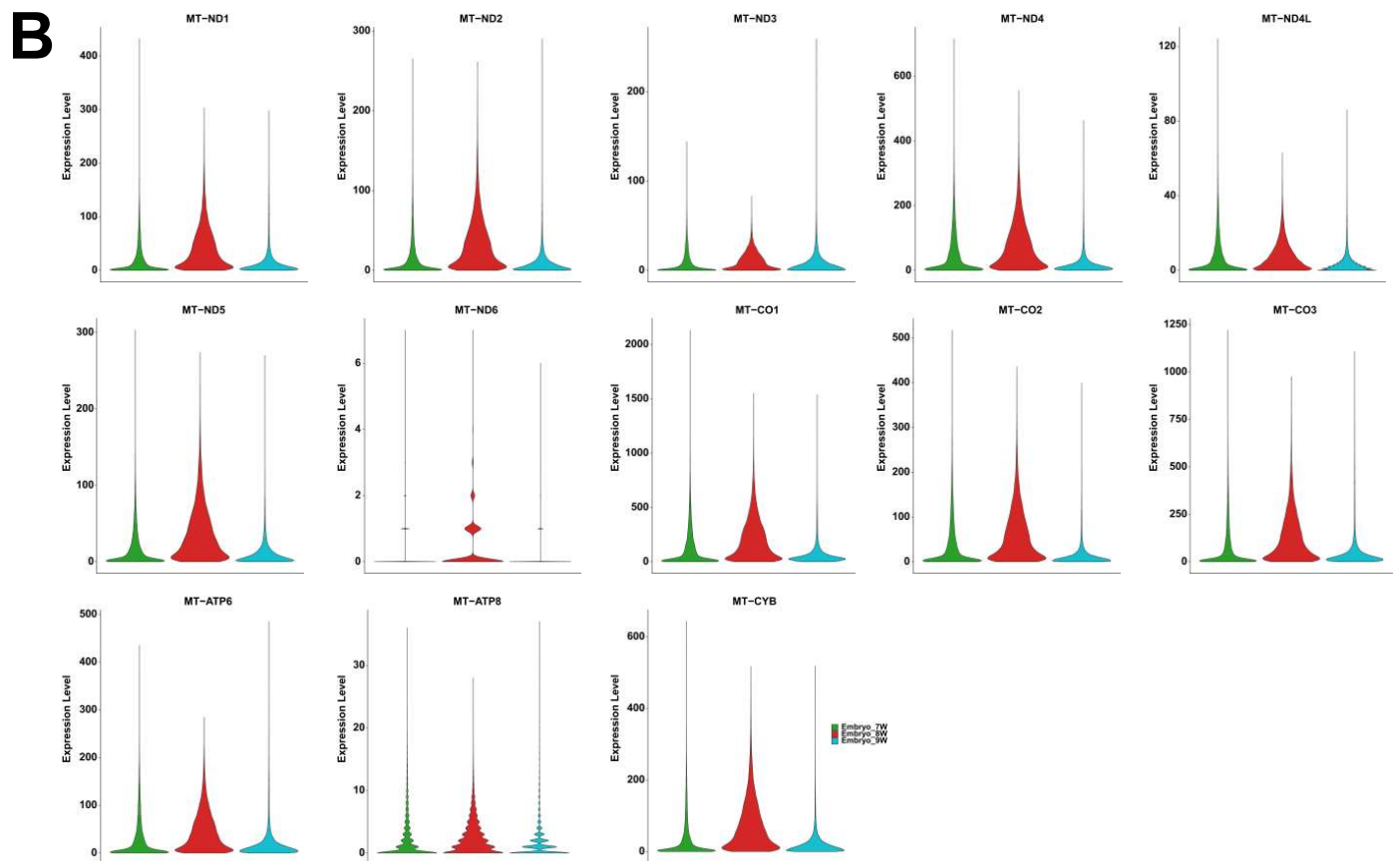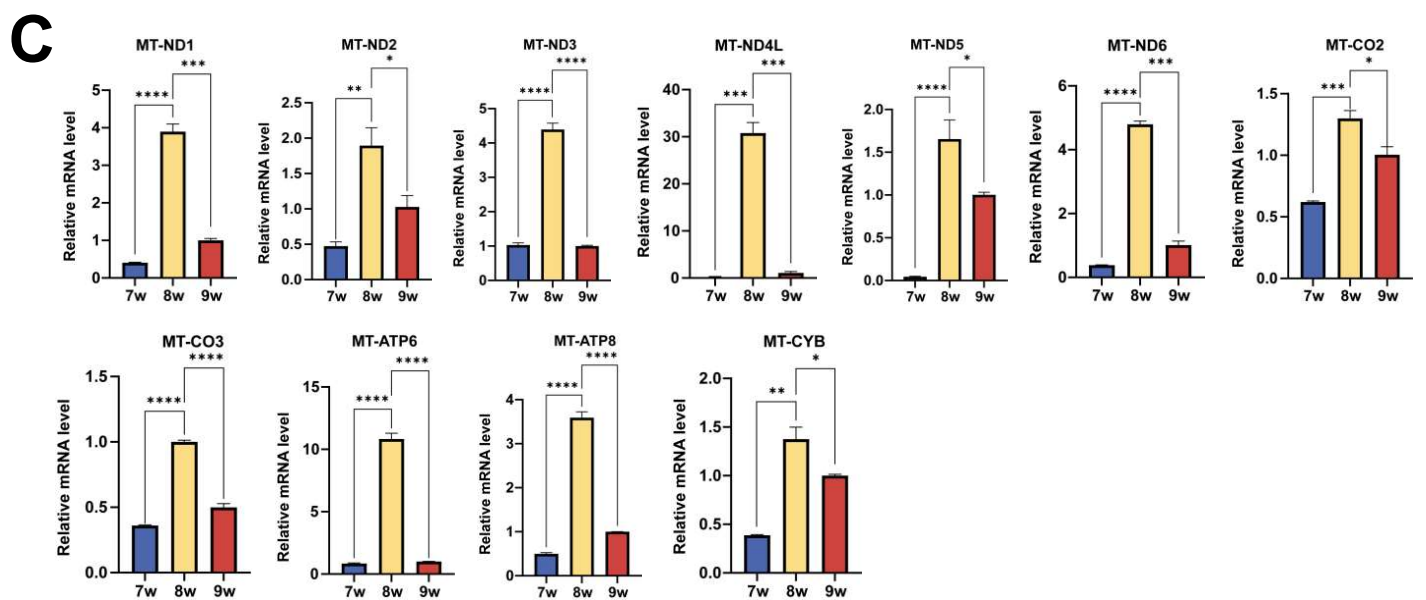

**Fig. S1**

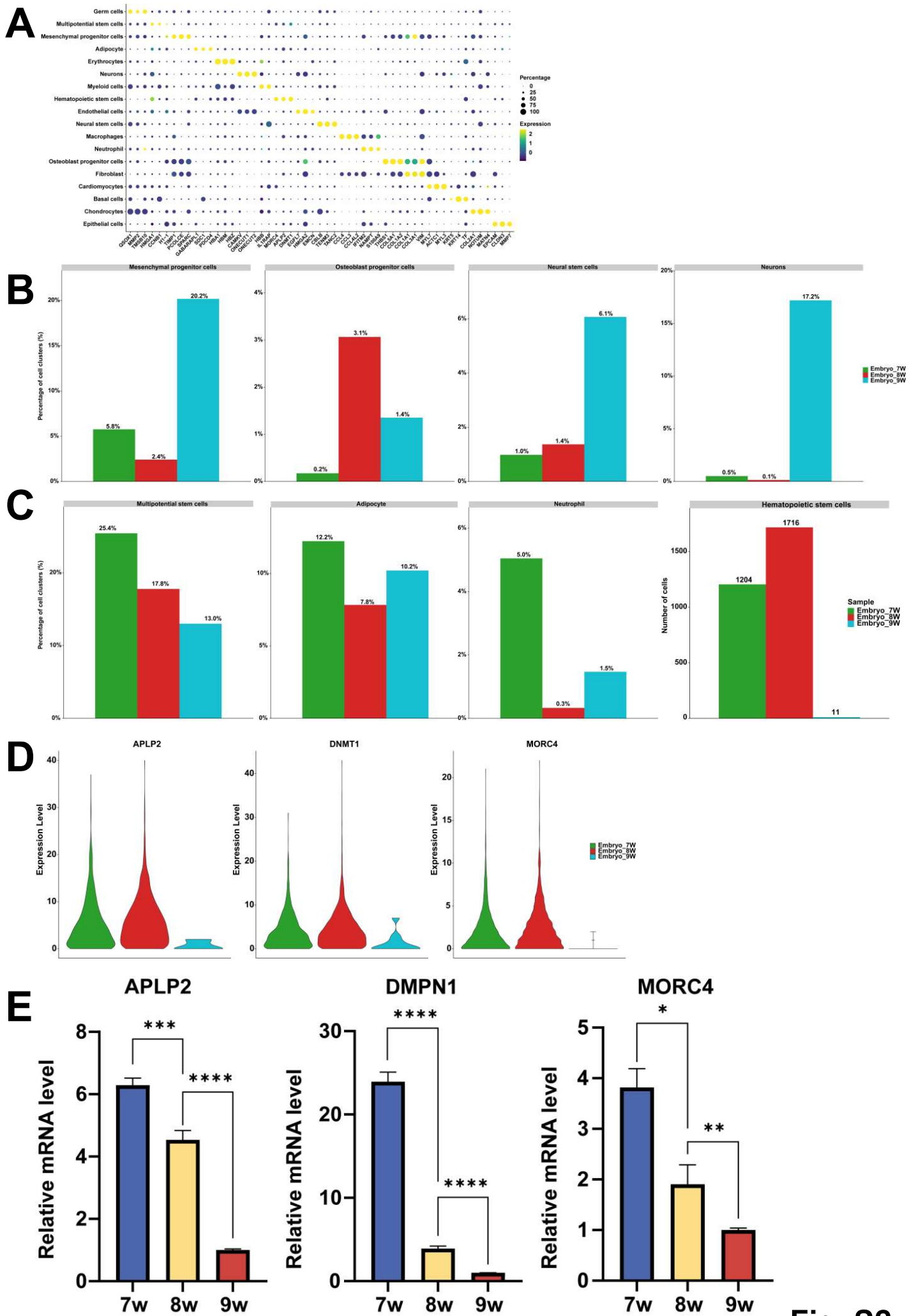

Fig. S2

**A**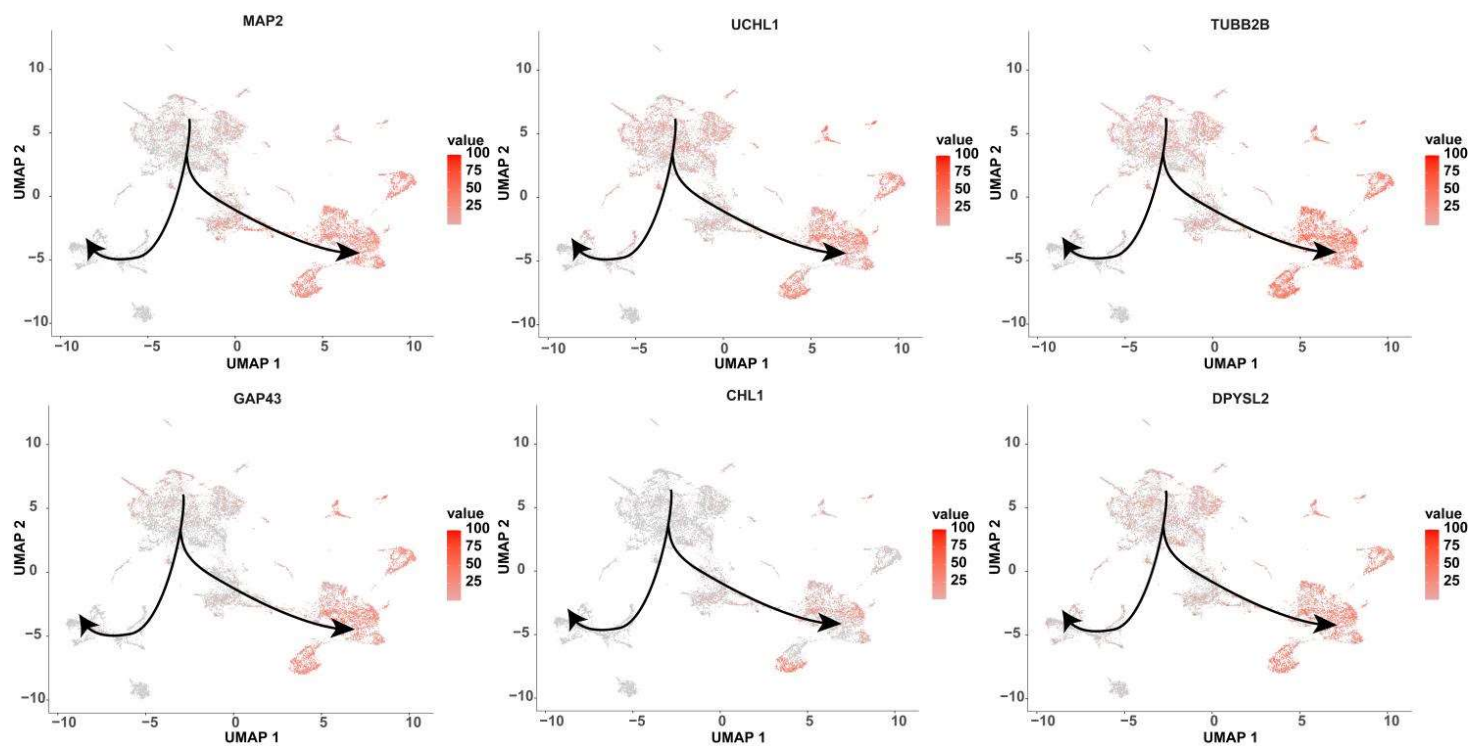**B**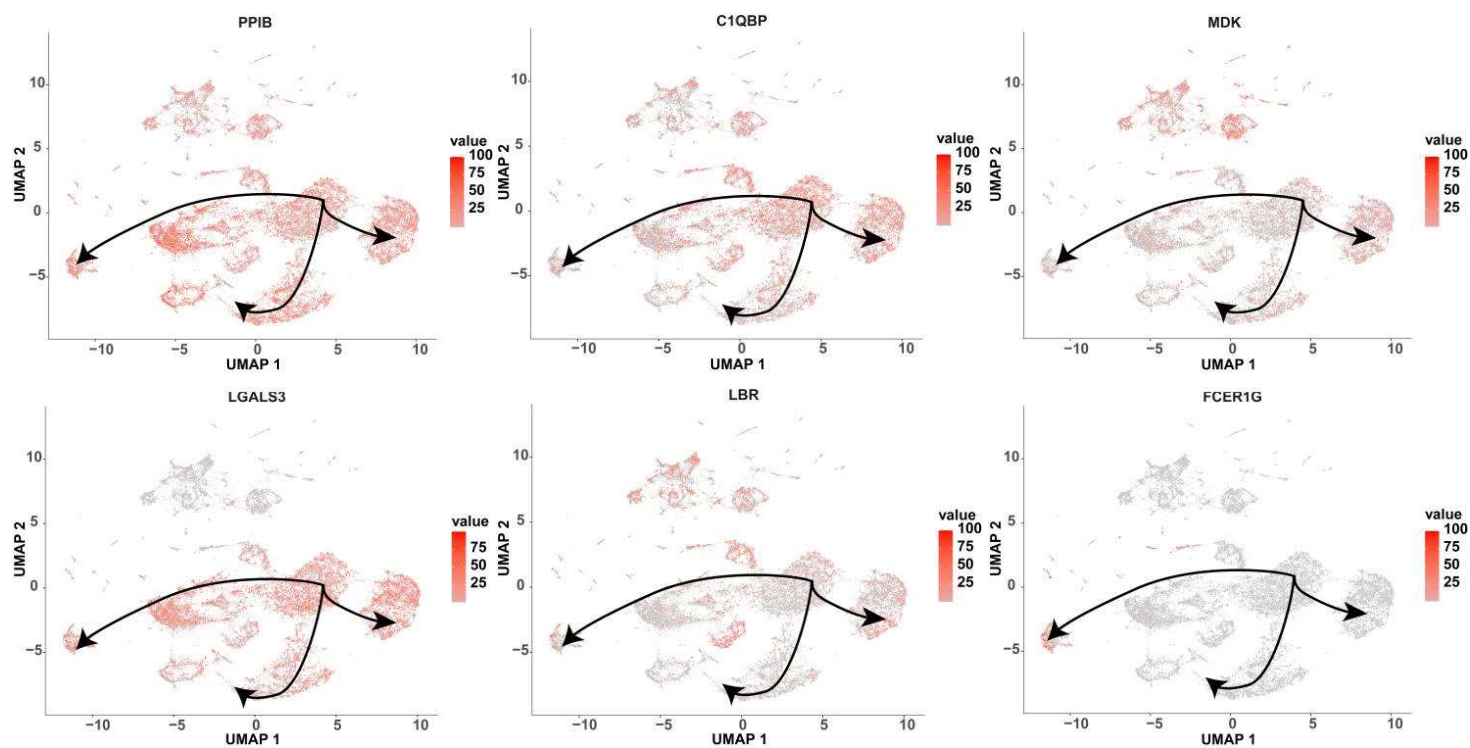**Fig. S3**

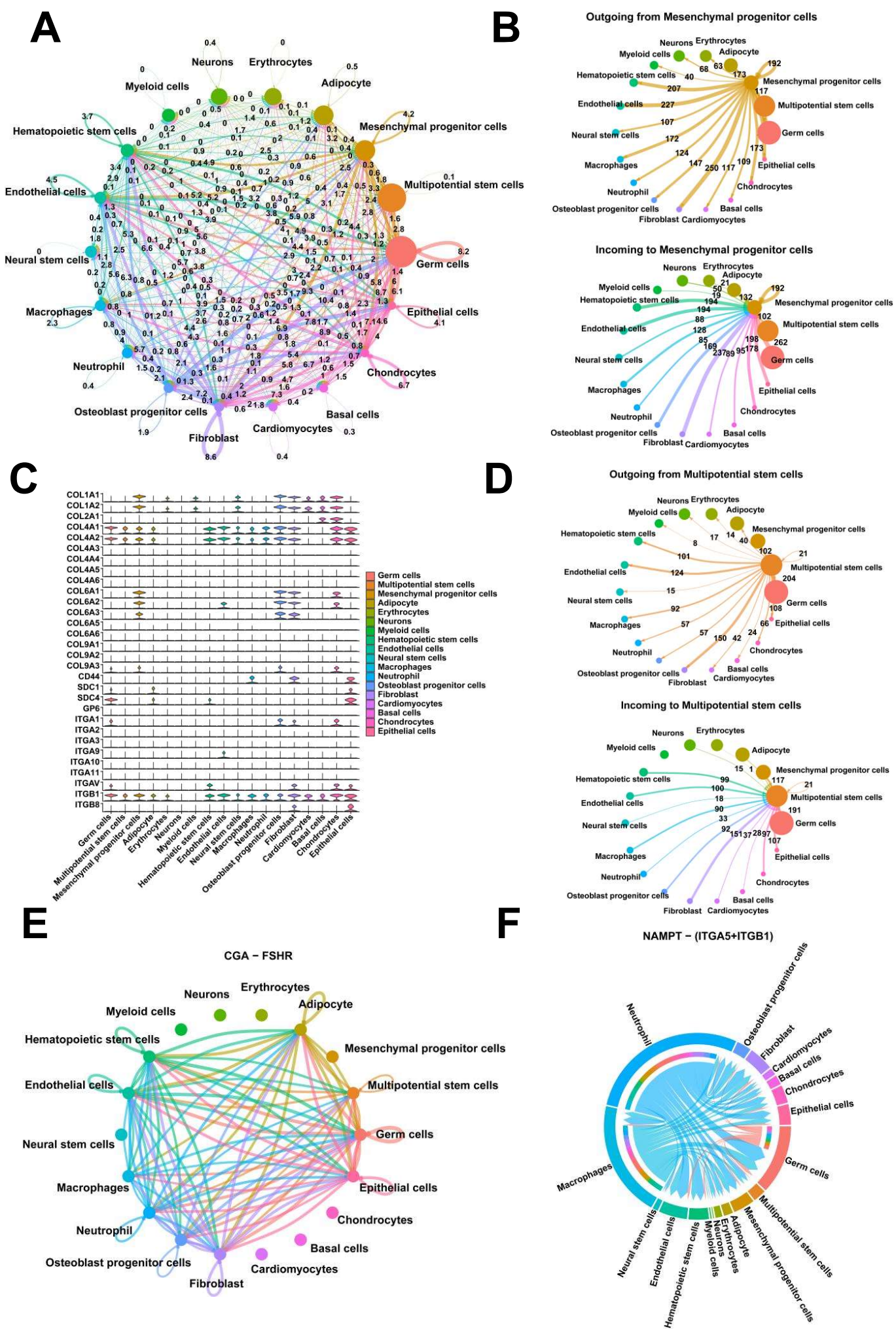

**Fig. S4**

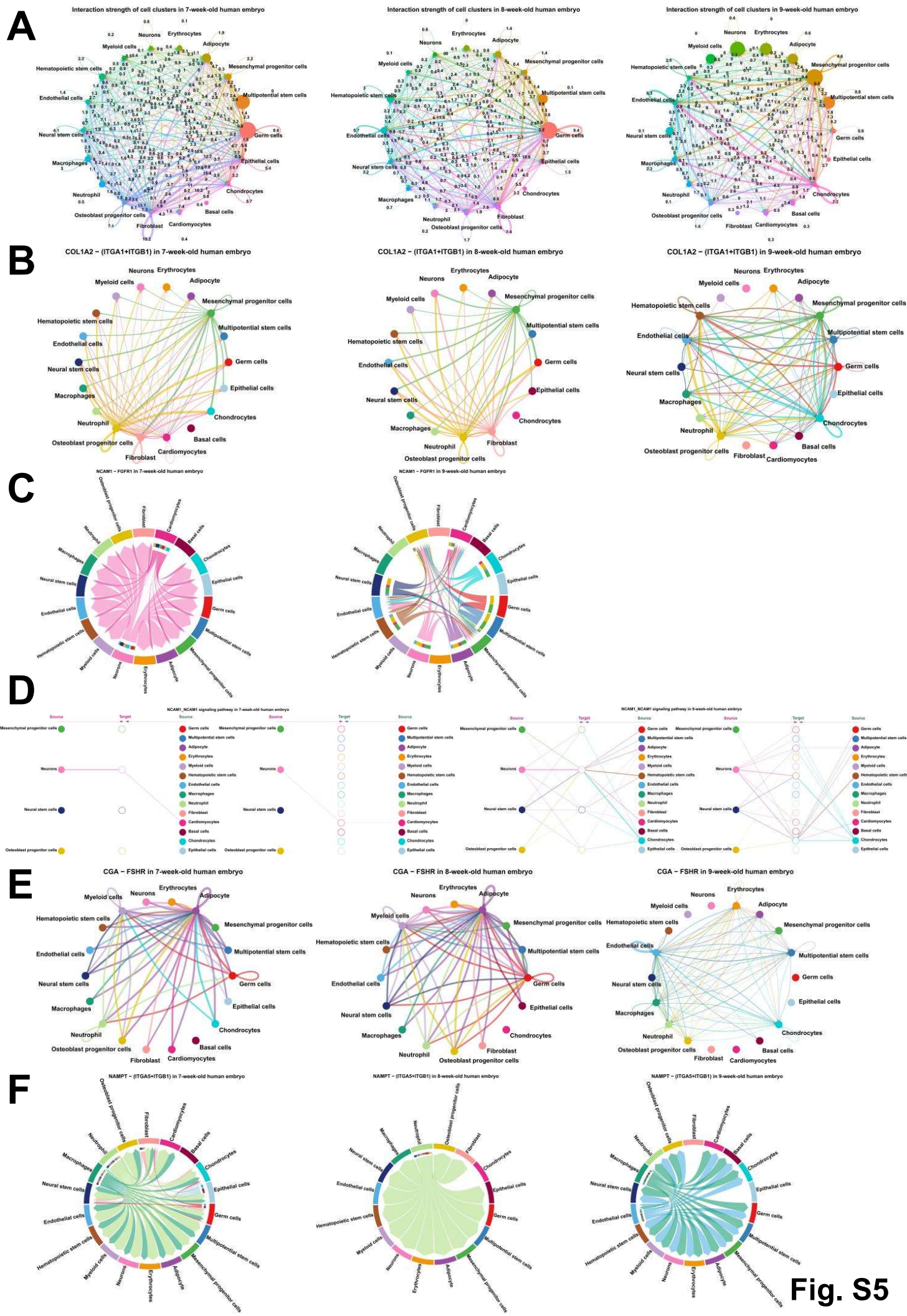

**Fig. S5**

Supplement: Supplementary file 1 — Additional file 1: Fig. S1. Quality control and expression of mitochondrial genes. A The number of genes, counts and percentage of mitochondria. B Violin plots showing the expression of mitochondrial genes including MT-ND1, MT-ND2, MT-CO1, MT-CO2, MT-ATP8, MT-ATP6, MT-CO3, MT-ND3, MT-ND4L, MT-ND4, MT-ND5, MT-ND6, MT-CYB in the different development stage of human embryos using single cell sequencing data. C qPCR validated the expression of mitochondrial genes in different development stages. Fig. S2. Marker genes and cell proportion analysis. A The marker genes which were screened from the published literatures. B The cell proportions of mesenchymal progenitor cells, osteoblast progenitor cells, neural stem cells and neurons. C The cell proportions of multipotential stem cells, adipocyte, neutrophil and the number of hematopoietic stem cells. D Violin plots showing the expression levels of MORC4, APLP2, DNMT1 in hematopoietic stem cells of different development stage of human embryos using single cell sequencing data. E qPCR validated the expression of MORC4, APLP2, DNMT1 in the different development stage of human embryos. Fig. S3. The expression of key genes along the pseudotime. A The expression of key genes including MAP2, UCHL1, TUBB2B, GAP43, CHL1 and DPYSL2 between mesenchymal progenitor cells and neural stem cells. B The expression of PPIB, C1QBP, MDK, LGALS3, LBR and FCER1G between multipotential stem cells and neutrophil. Fig. S4. Cell–Cell communication analysis of each cell cluster. A The interaction strength of ligand-receptors of each cell cluster. B The number of ligand-receptors of mesenchymal progenitor cells with other cell clusters. C The expression of COL1A2, ITGA1 and ITGB1 among 18 cell clusters. D The number of ligand-receptors of multipotential stem cells with other cell clusters. E CGA-FSHR signaling pathway in each cell cluster. F NAMPT-signaling pathway in each cell cluster. Fig. S5. The strength and signaling pathways of cell clusters in [file 13578_2024_1302_MOESM1_ESM.pdf]
