# Supplementary material for: Single‑cell RNA sequencing analysis of human embryos from the late Carnegie to fetal development
Source: Cell Biosci. 2024 Sep 12;14:118. doi: 10.1186/s13578-024-01302-9 (PMC11395182; doi:10.1186/s13578-024-01302-9)
Supplement: Supplementary file 2 — Additional file 2: Table S1. Marker genes for annotation of human embryo cell clusters. [file 13578_2024_1302_MOESM2_ESM.docx]

Table1 Marker genes for annotation of human embryo cell clusters

| 0 | Germ cells | QSOX1[1]  MMP2[2]  TMSB10[3]  S100A6[4] |
| --- | --- | --- |
| 1 | Multipotential stem cells | HMGA1[5]  CCNB1[6]  H1-1[7] |
| 2 | Mesenchymal progenitor cells | DKK1[8]  APOD[9]  TIMP1[10]  PCOLCE[11]  SPARC[12] |
| 3 | Adipocyte | APMAP[13]  GABARAPL1[14]  SDC1[15]  PDCD4[16]  PHOSPHO1[17] |
| 4 | Erythrocytes | HBA1[18]  HBA2[18]  HBG1[19]  HBM[20]  HBZ[21] |
| 5 | Neurons | CAMKV[22]  ONECUT1[23]  ONECUT2[23]  SNCG[24]  STMN1[25] |
| 6 | Myeloid cells | HBB[26]  IL1RAP[27] |
| **7** | Hematopoietic stem cells | MORC4[28]  APLP2[29]  DNMT1[30] |
| 8 | Endothelial cells | MMRN1[31]  PECAM1[32]  EGFL7[33]  HMGA2[34]  EMCN[35] |
| 9 | Neural stem cells | CBLB[36]  GRAMD1B[37]  TENM3[38]  TANC2[39]  MACF1[40] |
| 10 | Macrophages | CCL4[41]  CCL3[42]  CCL4L2[43]  CCL3L3[44]  PSAP[45] |
| 11 | Neutrophil | IFITM2[46]  NAMPT[47]  S100A9[48]  OLFM4[49] |
| 12 | Osteoblast progenitor cells | THBS1[50]  COL5A1[51]  COL1A2[52]  COL9A2[53] |
| 13 | Fibroblast | COL1A1[54]  COL3A1[54]  VIM[55]  LGALS1[56] |
| 14 | Cardiomyocytes | MYL7[57]  ACTC1[58]  MYL3[59]  TPM1[60]  LMOD2[61] |
| 15 | Basal cells | KRT5[62]  KRT14[63]  TF[63] |
| 16 | Chondrocytes | COL2A1[64]  NOTUM[65]  MATN4[66] |
| 17 | Epithelial cells | EPCAM[67]  CLDN3[68]  MMP7[69]  LCN2[70]  NUPR1[71] |

1. Wang TE, Li SH, Minabe S, Anderson AL, Dun MD, Maeda KI, et al. Mouse quiescin sulfhydryl oxidases exhibit distinct epididymal luminal distribution with segment-specific sperm surface associations. Biol Reprod*.* 2018; 99(5):1022-1033.

2. Yao PL, Lin YC, Richburg JH. TNF alpha-mediated disruption of spermatogenesis in response to Sertoli cell injury in rodents is partially regulated by MMP2. Biol Reprod*.* 2009; 80(3):581-589.

3. Turhan A, Pereira MT, Schuler G, Bleul U, Kowalewski MP. Hypoxia-inducible factor (HIF1alpha) inhibition modulates cumulus cell function and affects bovine oocyte maturation in vitrodagger. Biol Reprod*.* 2021; 104(2):479-491.

4. Ito M, Kizawa K, Hamada K, Cotsarelis G. Hair follicle stem cells in the lower bulge form the secondary germ, a biochemically distinct but functionally equivalent progenitor cell population, at the termination of catagen. Differentiation*.* 2004; 72(9-10):548-557.

5. Resar L, Chia L, Xian LL. Lessons from the Crypt: HMGA1-Amping up Wnt for Stem Cells and Tumor Progression. Cancer Res*.* 2018; 78(8):1890-1897.

6. Nguyen PD, Gurevich DB, Sonntag C, Hersey L, Alaei S, Nim HT, et al. Muscle Stem Cells Undergo Extensive Clonal Drift during Tissue Growth via Meox1-Mediated Induction of G2 Cell-Cycle Arrest. Cell Stem Cell*.* 2017; 21(1):107-119 e106.

7. Starkova TY, Artamonova TO, Ermakova VV, Chikhirzhina EV, Khodorkovskii MA, Tomilin AN. The Profile of Post-translational Modifications of Histone H1 in Chromatin of Mouse Embryonic Stem Cells. Acta Naturae*.* 2019; 11(2):82-91.

8. Mirfazeli ES, Arefian E, Nadri S, Rezazadeh Valojerdi R, Kehtari M, Zeynali B. DKK1 expression is suppressed by miR-9 during induced dopaminergic differentiation of human trabecular meshwork mesenchymal stem cells. Neurosci Lett*.* 2019; 707:134250.

9. Yu RH, Zhang XY, Xu W, Li ZK, Zhu XD. Apolipoprotein D alleviates glucocorticoid-induced osteogenesis suppression in bone marrow mesenchymal stem cells via the PI3K/Akt pathway. J Orthop Surg Res*.* 2020; 15(1):307.

10. Shu T, Zeng B, Ren XF, Li YL. HO-1 modified mesenchymal stem cells modulate MMPs/TIMPs system and adverse remodeling in infarcted myocardium. Tissue Cell*.* 2010; 42(4):217-222.

11. Li KX, Huo QJ, Dimmitt NH, Qu GF, Bao JJ, Pandya PH, et al. Osteosarcoma-enriched transcripts paradoxically generate osteosarcoma-suppressing extracellular proteins. Elife*.* 2023; 12.

12. Naito T, Yuge R, Kitadai Y, Takigawa H, Higashi Y, Kuwai T, et al. Mesenchymal stem cells induce tumor stroma formation and epithelial‑mesenchymal transition through SPARC expression in colorectal cancer. Oncol Rep*.* 2021; 45(6).

13. Haley SA, O'Hara BA, Atwood WJ. Adipocyte Plasma Membrane Protein (APMAP) promotes JC Virus (JCPyV) infection in human glial cells. Virology*.* 2020; 548:17-24.

14. Riera-Heredia N, Lutfi E, Balbuena-Pecino S, Velez EJ, Dias K, Beaumatin F, et al. The autophagy response during adipogenesis of primary cultured rainbow trout (Oncorhynchus mykiss) adipocytes. Comp Biochem Physiol B Biochem Mol Biol*.* 2022; 258:110700.

15. Zaragosi LE, Dadone B, Michiels JF, Marty M, Pedeutour F, Dani C, et al. Syndecan-1 regulates adipogenesis: new insights in dedifferentiated liposarcoma tumorigenesis. Carcinogenesis*.* 2015; 36(1):32-40.

16. Bai Y, Shang Q, Zhao H, Pan Z, Guo C, Zhang L, et al. Pdcd4 restrains the self-renewal and white-to-beige transdifferentiation of adipose-derived stem cells. Cell Death Dis*.* 2016; 7(3):e2169.

17. Jiang MX, Chavarria TE, Yuan BB, Lodish HF, Huang NJ. Phosphocholine accumulation and PHOSPHO1 depletion promote adipose tissue thermogenesis. Proc Natl Acad Sci U S A*.* 2020; 117(26):15055-15065.

18. Luo SQ, Chen XY, Tang N, Huang J, Zhong QY, Cai R, et al. Pedigree Analysis of Nonhomologous Sequence Recombination of HBA1 and HBA2 Genes. Hemoglobin*.* 2020; 44(5):329-333.

19. Wu YX, Zeng J, Roscoe BP, Liu PP, Yao QM, Lazzarotto CR, et al. Highly efficient therapeutic gene editing of human hematopoietic stem cells. Nat Med*.* 2019; 25(5):776-783.

20. Mi XQ, Chen JY, Zhou LW. Effect of low power laser irradiation on disconnecting the membrane-attached hemoglobin from erythrocyte membrane. J Photochem Photobiol B*.* 2006; 83(2):146-150.

21. Merryweather-Clarke AT, Tipping AJ, Lamikanra AA, Fa R, Abu-Jamous B, Tsang HP, et al. Distinct gene expression program dynamics during erythropoiesis from human induced pluripotent stem cells compared with adult and cord blood progenitors. BMC Genomics*.* 2016; 17(1):817.

22. Liang ZY, Zhan Y, Shen Y, Wong CCL, Yates JR, 3rd, Plattner F, et al. The pseudokinase CaMKv is required for the activity-dependent maintenance of dendritic spines. Nat Commun*.* 2016; 7:13282.

23. Klimova L, Antosova B, Kuzelova A, Strnad H, Kozmik Z. Onecut1 and Onecut2 transcription factors operate downstream of Pax6 to regulate horizontal cell development. Dev Biol*.* 2015; 402(1):48-60.

24. Liu JY, Shao T, Zhang J, Liu QY, Hua H, Zhang HY, et al. Gamma synuclein promotes cancer metastasis through the MKK3/6-p38MAPK cascade. Int J Biol Sci*.* 2022; 18(8):3167-3177.

25. Ding XL, Hu J, Zhang HY, Xu YX. Genetic Variants in the STMN1 Transcriptional Regulatory Region Affect Promoter Activity and Fear Behavior in English Springer Spaniels. PLoS One*.* 2016; 11(7):e0158756.

26. Luo P, Liu XY, Tang ZH, Xiong B. Decreased expression of HBA1 and HBB genes in acute myeloid leukemia patients and their inhibitory effects on growth of K562 cells. Hematology*.* 2022; 27(1):1003-1009.

27. Landberg N, Hansen N, Askmyr M, Agerstam H, Lassen C, Rissler M, et al. IL1RAP expression as a measure of leukemic stem cell burden at diagnosis of chronic myeloid leukemia predicts therapy outcome. Leukemia*.* 2016; 30(1):253-257.

28. Noren E, Verma D, Soderkvist P, Weisselberg T, Soderman J, Lotfi K, et al. Single Nucleotide Polymorphisms in MORC4, CD14, and TLR4 Are Related to Outcome of Allogeneic Stem Cell Transplantation. Ann Transplant*.* 2016; 21:56-67.

29. Mirinics ZK, Calafat J, Udby L, Lovelock J, Kjeldsen L, Rothermund K, et al. Identification of the presenilins in hematopoietic cells with localization of presenilin 1 to neutrophil and platelet granules. Blood Cells Mol Dis*.* 2002; 28(1):28-38.

30. Ahmadnejad M, Amirizadeh N, Mehrasa R, Karkhah A, Nikougoftar M, Oodi A. Elevated expression of DNMT1 is associated with increased expansion and proliferation of hematopoietic stem cells co-cultured with human MSCs. Blood Res*.* 2017; 52(1):25-30.

31. Parker DN, Tasneem S, Farndale RW, Bihan D, Sadler JE, Sebastian S, et al. The functions of the A1A2A3 domains in von Willebrand factor include multimerin 1 binding. Thromb Haemost*.* 2016; 116(1):87-95.

32. Hu DH, Zhang L, Yang F, Chen FX, Li DQ, Cai CL. Generation of Pecam1 endothelial specific dual reporter mouse model. Genesis*.* 2020; 58(9):e23384.

33. d'Audigier C, Susen S, Blandinieres A, Mattot V, Saubamea B, Rossi E, et al. Egfl7 Represses the Vasculogenic Potential of Human Endothelial Progenitor Cells. Stem Cell Rev Rep*.* 2018; 14(1):82-91.

34. Li DK, Chen XR, Wang LN, Wang JH, Li JK, Zhou ZY, et al. Exosomal HMGA2 protein from EBV-positive NPC cells destroys vascular endothelial barriers and induces endothelial-to-mesenchymal transition to promote metastasis. Cancer Gene Ther*.* 2022; 29(10):1439-1451.

35. Zhu Y, Ruan Z, Lin ZY, Long HT, Zhao RB, Sun BH, et al. The association between CD31(hi)Emcn(hi) endothelial cells and bone mineral density in Chinese women. J Bone Miner Metab*.* 2019; 37(6):987-995.

36. Wang Y, Lai XW, Wu DP, Liu B, Wang NX, Rong LM. Umbilical mesenchymal stem cell-derived exosomes facilitate spinal cord functional recovery through the miR-199a-3p/145-5p-mediated NGF/TrkA signaling pathway in rats. Stem Cell Res Ther*.* 2021; 12(1):117.

37. Esposito F, Osiceanu AM, Sorosina M, Ottoboni L, Bollman B, Santoro S, et al. A Whole-Genome Sequencing Study Implicates GRAMD1B in Multiple Sclerosis Susceptibility. Genes (Basel)*.* 2022; 13(12).

38. Antinucci P, Suleyman O, Monfries C, Hindges R. Neural Mechanisms Generating Orientation Selectivity in the Retina. Curr Biol*.* 2016; 26(14):1802-1815.

39. Guo H, Bettella E, Marcogliese PC, Zhao RJ, Andrews JC, Nowakowski TJ, et al. Disruptive mutations in TANC2 define a neurodevelopmental syndrome associated with psychiatric disorders. Nat Commun*.* 2019; 10(1):4679.

40. Moffat JJ, Ka MH, Jung EM, Smith AL, Kim WY. The role of MACF1 in nervous system development and maintenance. Semin Cell Dev Biol*.* 2017; 69:9-17.

41. Mukaida N, Sasaki SI, Baba T. CCL4 Signaling in the Tumor Microenvironment. Adv Exp Med Biol*.* 2020; 1231:23-32.

42. Sheng DD, Ma W, Zhang R, Zhou L, Deng QD, Tu J, et al. Ccl3 enhances docetaxel chemosensitivity in breast cancer by triggering proinflammatory macrophage polarization. J Immunother Cancer*.* 2022; 10(5).

43. Li XY, Sun H, Li H, Li D, Cai ZQ, Xu J, et al. A Single-Cell RNA-Sequencing Analysis of Distinct Subsets of Synovial Macrophages in Rheumatoid Arthritis. DNA Cell Biol*.* 2023; 42(4):212-222.

44. Dzakah EE, Zhao JC, Wang LY, Rashid F, Xu R, Yang LG, et al. Chlamydia trachomatis Stimulation Enhances HIV-1 Susceptibility through the Modulation of a Member of the Macrophage Inflammatory Proteins. J Invest Dermatol*.* 2022; 142(5):1338-1348 e1336.

45. Taoerdahong H, Zhou K, Yang F, Dong CX. Structure, immunostimulatory activity, and the effect of ameliorating airway inflammation of polysaccharides from Pyrus sinkiangensis Yu. Int J Biol Macromol*.* 2022; 195:246-254.

46. Eddins DJ, Yang JK, Kosters A, Giacalone VD, Pechuan-Jorge X, Chandler JD, et al. Transcriptional reprogramming of infiltrating neutrophils drives lung pathology in severe COVID-19 despite low viral load. Blood Adv*.* 2023; 7(5):778-799.

47. Siakaeva E, Pylaeva E, Spyra I, Bordbari S, Hoing B, Kurten C, et al. Neutrophil Maturation and Survival Is Controlled by IFN-Dependent Regulation of NAMPT Signaling. Int J Mol Sci*.* 2019; 20(22).

48. Mellor LF, Gago-Lopez N, Bakiri L, Schmidt FN, Busse B, Rauber S, et al. Keratinocyte-derived S100A9 modulates neutrophil infiltration and affects psoriasis-like skin and joint disease. Ann Rheum Dis*.* 2022; 81(10):1400-1408.

49. Bonaventura A, Montecucco F, Dallegri F, Carbone F, Luscher TF, Camici GG, et al. Novel findings in neutrophil biology and their impact on cardiovascular disease. Cardiovasc Res*.* 2019; 115(8):1266-1285.

50. Shi JW, Zhou YW, Chen YF, Ye M, Qiao F, Tian JW, et al. Epididymis cell atlas in a patient with a sex development disorder and a novel NR5A1 gene mutation. Asian J Androl*.* 2023; 25(1):103-112.

51. Kahai S, Vary CP, Gao YG, Seth A. Collagen, type V, alpha1 (COL5A1) is regulated by TGF-beta in osteoblasts. Matrix Biol*.* 2004; 23(7):445-455.

52. Maruelli S, Besio R, Rousseau J, Garibaldi N, Amiaud J, Brulin B, et al. Osteoblasts mineralization and collagen matrix are conserved upon specific Col1a2 silencing. Matrix Biol Plus*.* 2020; 6-7:100028.

53. Lopez-Cuevas P, Deane L, Yang YS, Hammond CL, Kague E. Transformed notochordal cells trigger chronic wounds in zebrafish, destabilizing the vertebral column and bone homeostasis. Dis Model Mech*.* 2021; 14(3).

54. Tao R, Fan XX, Yu HJ, Ai G, Zhang HY, Kong HY, et al. MicroRNA-29b-3p prevents Schistosoma japonicum-induced liver fibrosis by targeting COL1A1 and COL3A1. J Cell Biochem*.* 2018; 119(4):3199-3209.

55. Cheng F, Shen Y, Mohanasundaram P, Lindstrom M, Ivaska J, Ny T, et al. Vimentin coordinates fibroblast proliferation and keratinocyte differentiation in wound healing via TGF-beta-Slug signaling. Proc Natl Acad Sci U S A*.* 2016; 113(30):E4320-4327.

56. Tsai YT, Li CY, Huang YH, Chang TS, Lin CY, Chuang CH, et al. Galectin-1 orchestrates an inflammatory tumor-stroma crosstalk in hepatoma by enhancing TNFR1 protein stability and signaling in carcinoma-associated fibroblasts. Oncogene*.* 2022; 41(21):3011-3023.

57. Sun JM, Guo XP, Yu P, Liang JN, Mo ZX, Zhang MY, et al. Vasorin deficiency leads to cardiac hypertrophy by targeting MYL7 in young mice. J Cell Mol Med*.* 2022; 26(1):88-98.

58. Jiang HK, Qiu GR, Li-Ling J, Xin N, Sun KL. Reduced ACTC1 expression might play a role in the onset of congenital heart disease by inducing cardiomyocyte apoptosis. Circ J*.* 2010; 74(11):2410-2418.

59. Berge KE, Leren TP. Genetics of hypertrophic cardiomyopathy in Norway. Clin Genet*.* 2014; 86(4):355-360.

60. Sewanan LR, Park J, Rynkiewicz MJ, Racca AW, Papoutsidakis N, Schwan J, et al. Loss of crossbridge inhibition drives pathological cardiac hypertrophy in patients harboring the TPM1 E192K mutation. J Gen Physiol*.* 2021; 153(9).

61. Lay E, Azamian MS, Denfield SW, Dreyer W, Spinner JA, Kearney D, et al. LMOD2-related dilated cardiomyopathy presenting in late infancy. Am J Med Genet A*.* 2022; 188(6):1858-1862.

62. Kathiriya JJ, Wang CQ, Zhou MQ, Brumwell A, Cassandras M, Le Saux CJ, et al. Human alveolar type 2 epithelium transdifferentiates into metaplastic KRT5(+) basal cells. Nat Cell Biol*.* 2022; 24(1):10-23.

63. Rock JR, Onaitis MW, Rawlins EL, Lu Y, Clark CP, Xue Y, et al. Basal cells as stem cells of the mouse trachea and human airway epithelium. Proc Natl Acad Sci U S A*.* 2009; 106(31):12771-12775.

64. McAlinden A, Johnstone B, Kollar J, Kazmi N, Hering TM. Expression of two novel alternatively spliced COL2A1 isoforms during chondrocyte differentiation. Matrix Biol*.* 2008; 27(3):254-266.

65. Isojima T, Sims NA. Cortical bone development, maintenance and porosity: genetic alterations in humans and mice influencing chondrocytes, osteoclasts, osteoblasts and osteocytes. Cell Mol Life Sci*.* 2021; 78(15):5755-5773.

66. Li P, Fleischhauer L, Nicolae C, Prein C, Farkas Z, Saller MM, et al. Mice Lacking the Matrilin Family of Extracellular Matrix Proteins Develop Mild Skeletal Abnormalities and Are Susceptible to Age-Associated Osteoarthritis. Int J Mol Sci*.* 2020; 21(2).

67. Lee CC, Yu CJ, Panda SS, Chen KC, Liang KH, Huang WC, et al. Epithelial cell adhesion molecule (EpCAM) regulates HGFR signaling to promote colon cancer progression and metastasis. J Transl Med*.* 2023; 21(1):530.

68. Che JJ, Yue DS, Zhang B, Zhang H, Huo YS, Gao LW, et al. Claudin-3 Inhibits Lung Squamous Cell Carcinoma Cell Epithelial-mesenchymal Transition and Invasion via Suppression of the Wnt/beta-catenin Signaling Pathway. Int J Med Sci*.* 2018; 15(4):339-351.

69. Zhang Q, Liu S, Parajuli KR, Zhang W, Zhang K, Mo Z, et al. Interleukin-17 promotes prostate cancer via MMP7-induced epithelial-to-mesenchymal transition. Oncogene*.* 2017; 36(5):687-699.

70. Gupta U, Ghosh S, Wallace CT, Shang P, Xin Y, Nair AP, et al. Increased LCN2 (lipocalin 2) in the RPE decreases autophagy and activates inflammasome-ferroptosis processes in a mouse model of dry AMD. Autophagy*.* 2023; 19(1):92-111.

71. Zhou RM, Liao JS, Cai DP, Tian Q, Huang EP, Lu TM, et al. Nupr1 mediates renal fibrosis via activating fibroblast and promoting epithelial-mesenchymal transition. FASEB J*.* 2021; 35(3):e21381.
