# Supplementary material for: Single‑cell RNA sequencing analysis of human embryos from the late Carnegie to fetal development
Source: Cell Biosci. 2024 Sep 12;14:118. doi: 10.1186/s13578-024-01302-9 (PMC11395182; doi:10.1186/s13578-024-01302-9)
Supplement: Supplementary file 3 — Additional file 3: Table S2. Primers for quantitative RT-PCR. [file 13578_2024_1302_MOESM3_ESM.docx]

Table2 Primers for quantitative RT-PCR

| Genes | Forward primer sequence (5’- 3’) | Reverse primer sequence （5’ - 3’） |
| --- | --- | --- |
| MT-ND1 | CCCTAAAACCCGCCACATCT | GAGCGATGGTGAGAGCTAAGGT |
| MT-ND2 | AACCCTCGTTCCACAGAAGCT | AGGAGAAGGCTTACGTTTAGT |
| MT-ND3 | ACCACAACTCAACGGCTACA | CCAGACTTAGGGCTAGGATGATG |
| MT-ND4L | TCTGGCCTATGAGTGACTAC | AGTATTATTCCTTCTAGGCA |
| MT-ND5 | CAGCCCTACTCCACTCAAGC | TAGTAAGGGTGGGGAAGCGA |
| MT-ND6 | ACACTCAACAAGACCTCAACC | TAGTTTTTTTAATTTATTTAGGGGGACT |
| MT-CO2 | CTGAACCTACGAGTACACCG | TTAATTCTAGGACGATGGGC |
| MT-CO3 | CACATAATGACCCACCAATCA | AATAAGCAGTGCTTGAATTAT |
| MT-ATP6 | CGCCACCCTAGCAATATCAA | TTAAGGCGACAGCGATTTCT |
| MT-ATP8 | ATTAATTCCCCTAAAAATCT | AATGAATGAAGCGAACAGAT |
| MT-CYB | GGGGCCACAGTAATTACAAA | GGGGGTTGTTTGATCCCGTTT |
| APLP2 | TGAGCCTCAAATCGCAATGTT | CCTGTTGGATCAGGTTCCCAT |
| MORC4 | AGGTGACTACCCAGATGATTGC | CCTTCTCCACGAGTTGGCTT |
| DNMT1 | AGAACGGTGCTCATGCTTACA | CTCTACGGGCTTCACTTCTTG |
| ACTIN | TTGTTACAGGAAGTCCCTTGCC | ATGCTATCACCTCCCCTGTGTG |
